# Molecular Basis of Prostate Cancer and Natural Products as Potential Chemotherapeutic and Chemopreventive Agents

**DOI:** 10.3389/fphar.2021.738235

**Published:** 2021-09-23

**Authors:** Bingke Bai, Qianbo Chen, Rui Jing, Xuhui He, Hongrui Wang, Yanfei Ban, Qi Ye, Weiheng Xu, Chengjian Zheng

**Affiliations:** ^1^ Department of Chinese Medicine Authentication, School of Pharmacy, Second Military Medical University, Shanghai, China; ^2^ Department of Anesthesiology, Shanghai Eastern Hepatobiliary Surgery Hospital, Shanghai, China; ^3^ Department of Biological Science, College of Life Science, Fujian Agriculture and Forestry University, Fuzhou, China; ^4^ Department of Biochemical Pharmacy, School of Pharmacy, Second Military Medical University, Shanghai, China

**Keywords:** prostate cancer, natural compounds, apoptosis, androgen receptor, mechanism

## Abstract

Prostate cancer is the second most common malignant cancer in males. It involves a complex process driven by diverse molecular pathways that closely related to the survival, apoptosis, metabolic and metastatic characteristics of aggressive cancer. Prostate cancer can be categorized into androgen dependent prostate cancer and castration-resistant prostate cancer and cure remains elusive due to the developed resistance of the disease. Natural compounds represent an extraordinary resource of structural scaffolds with high diversity that can offer promising chemical agents for making prostate cancer less devastating and curable. Herein, those natural compounds of different origins and structures with potential cytotoxicity and/or *in vivo* anti-tumor activities against prostate cancer are critically reviewed and summarized according to the cellular signaling pathways they interfere. Moreover, the anti-prostate cancer efficacy of many nutrients, medicinal plant extracts and Chinese medical formulations were presented, and the future prospects for the application of these compounds and extracts were discussed. Although the failure of conventional chemotherapy as well as involved serious side effects makes natural products ideal candidates for the treatment of prostate cancer, more investigations of preclinical and even clinical studies are necessary to make use of these medical substances reasonably. Therefore, the elucidation of structure-activity relationship and precise mechanism of action, identification of novel potential molecular targets, and optimization of drug combination are essential in natural medicine research and development.

## Introduction

It is nowadays evident that prostate cancer (PCa) is recognized as the second most common cancer and the fifth cause of cancer death in males (Torre et al., 2015)Initially, the usual therapy for prostate cancer is prostatectomy or radiation, which aims to remove or kill the malignant cells that have not spread or metastasized (Feldman and Feldman, 2001). However, numerous patients cannot be cured thoroughly by this treatment, and then followed by cancer recurrence and/or metastasis. The majority of prostate cancer growth is androgen dependent. Androgen deprivation therapy (ADT) such as surgery or gonadotropin-releasing hormone (GnRH) analog treatment, is the main therapeutic and dramatically effective intervention for the treatment of androgen dependent prostate cancer (ADPC) in putting patients with tumors in remission, as documented by the work on castration of Huggins, who was awarded the Nobel prize in 1966 (Huggins, 1978). Nevertheless, after this therapy, most of these prostate cancer patients gradually become androgen independent, go on to progress, metastasize and resist to ADT within 13–24 months accompanied by increased levels of prostate-specific antigen (PSA). Siegel et al. reported that failure of ADT is responsible for the ∼27,000 metastatic prostate cancer deaths in the United States annually (Siegel et al., 2017). This stage of prostate cancer is called castration-resistant prostate cancer (CRPC), which has poor prognosis (Small et al., 2004). At present, there is no effective therapy for CRPC besides docetaxel, which has been demonstrated to prolong overall survival in this patient population. However, the efficacy of docetaxel is not satisfactory and there are many severe adverse effects such as anemia, neutropenia, diarrhea, and sensory neuropathy. Although, therapeutic options have expanded rapidly since 2011, including AR inhibitors (enzalutamide, abiraterone), immunotherapy (sipuleucel-T), bone seeking radionuclides (radium-223), and second-line chemotherapy (cabazitaxel), all of these agents or interventions only have shown a median survival benefit of 2–5 months (Ritch and Cookson, 2016). So searching for more effective anti-prostate cancer drugs, especially with high efficacy and low toxicity, remains an urgent problem that needs to be resolved. Natural compounds represent an irreplaceable resource of structural scaffolds that can offer chemical agents for making prostate cancer less devastating and curable. In recent years, many natural products and extracts have been scientifically investigated *in vitro* and/or *in vivo* and proved as potential anti-prostate cancer agents, which are currently scattered across various publications. So a systematic summary and knowledge of future prospects are necessary to facilitate further chemical and pharmacological studies for anti-prostate cancer agents.

Herein, we reviewed the detailed molecular causes of prostate cancer and critically summarized the natural compounds (or extracts and Chinese herb preparations) that have been reported to inhibit prostate cancer cells proliferation/tumor growth, induce prostate cancer cells apoptosis or exhibit effects on specific signaling pathways involved in prostate cancer *in vivo* and *in vitro*. In addition, we also provided possible novel targets for screening natural compounds (or extracts or Chinese herb preparations) with anti-prostate cancer activity and discuss the future prospects for the application of these compounds and extracts and the novel available approaches and technological improvements that should be explored to treat prostate cancer.

## Overview of the Molecular Basis of Prostate Cancer

### Molecular Basis of Androgen Dependent Prostate Cancer

Androgens, principally testosterone (T) and dihydrotestosterone (DHT), are synthesized from cholesterol as the initial 27-carbon substrate via multiple enzymatic steps (Wadosky and Koochekpour, 2016). As a member of the ligand-activated nuclear hormone receptors superfamily, androgen receptor (AR) is a modular protein with four functional domains: an N-terminal regulatory domain (NTD), a DNA-binding domain (DBD), a small hinge region (H) and a ligand-binding domain (LBD) (Ho and Dehm, 2017). Upon binding to androgens, AR undergoes a conformational change, leading to nuclear translocation, phosphorylation, homodimer, and interaction with DNA (Li and Al-Azzawi, 2009). Subsequently, AR dimer binds to androgen-response elements (AREs), recruits essential co-factors and regulates the expression of androgen-regulated genes (Ho and Dehm, 2017).

The development and maintenance of the prostate is inseparable from androgen acting through the AR. Since Huggins and Hodges first demonstrated the responsiveness of prostate cancer to androgen deprivation, it has been clear that prostate cancer is dependent on androgen and AR activation for growth and survival (Huggins and Hodges, 1941). From then, hundreds of studies have demonstrated that androgen withdrawal results in initial regression of essentially all prostate cancers, albeit for a finite period, with the ultimate development of castration-resistant disease. Androgen deprivation therapy, via either orchidectomy or use of a gonadotropin-releasing hormone (GnRH) agonist has become the cornerstone of therapy in the treatment of prostate cancers. Newer agents, such as abiraterone, which block androgen synthetic pathways, have added clinical benefit in disseminated disease, demonstrating that even in “castration resistant disease” androgens may still be supporting prostate cancer growth (Morgentaler, 2009). These data support the notion that prostate cancer, in most cases, is a hormone (androgen) sensitive disease.

### Overview of the Mechanisms of Castration-Resistant Prostate Cancer

Historically, there are much debate about the mechanisms of castration resistance, which are mainly summarized as the following by recent studies: canonical AR signaling, relying on AR nuclear translocation and AR-DNA binding, and non-nuclear AR signaling which requires neither AR nuclear translocation nor AR-DNA binding(Qin and Bin, 2019; Pisano et al., 2021).

#### Canonical AR Signaling

**FIGURE 1:**
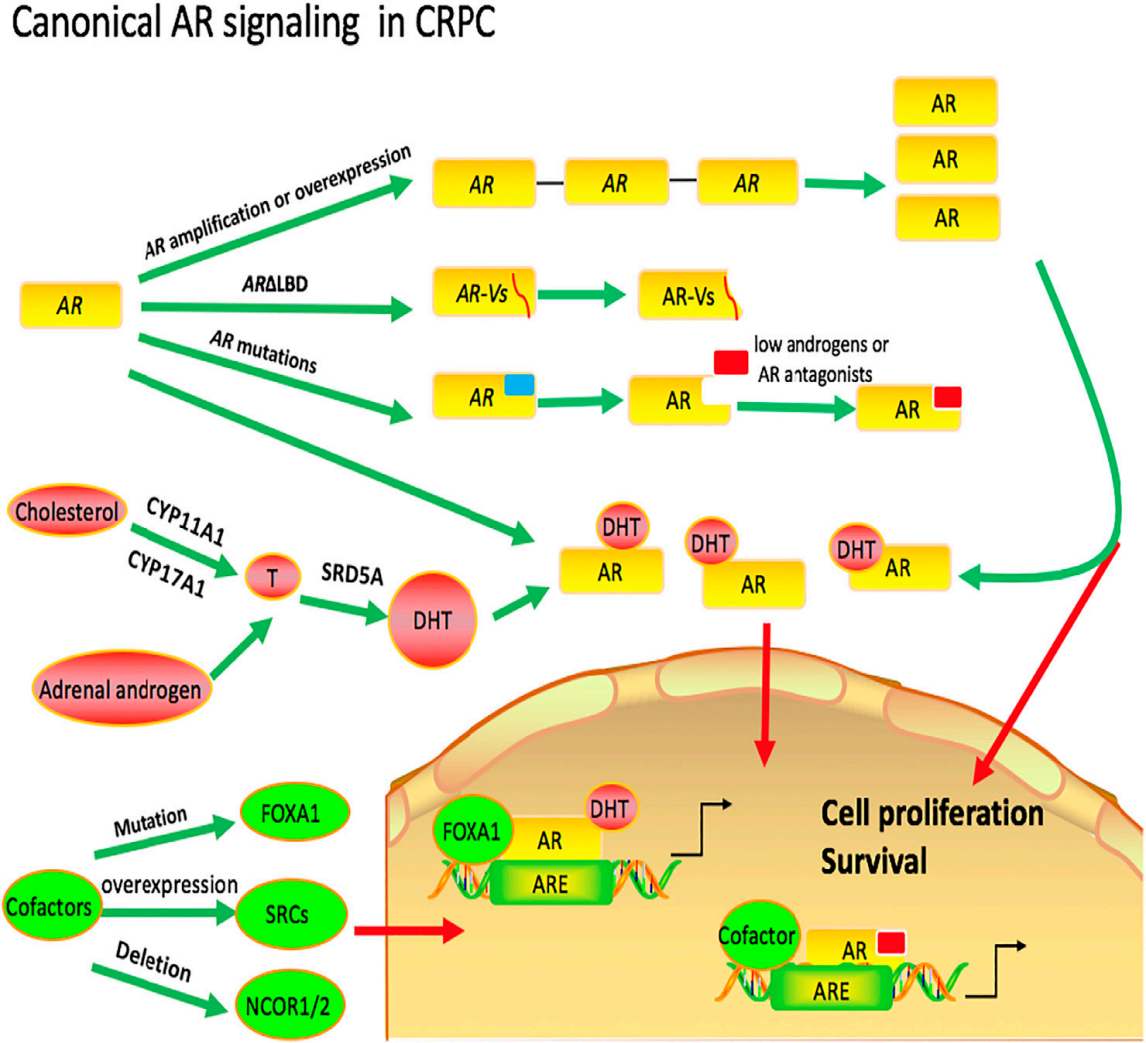
Canonical AR in castration-resistant prostate cancer. Common alterations in *AR*, including *AR* amplification or overexpression, *AR* mutations and truncated *AR* lacking ligand-binding domains (*AR*ΔLBD), can increase its sensitivity to very low levels of androgens or lead to constitutive activation of AR signaling. There are two possible ways for the initial substrates to convert to intratumoral DHT in CRPC. The first way is that androgens are synthesized intratumorally via the *de novo* route and the second is that androgens are converted from adrenal androgens. Genomic and transcriptomic alterations in AR pathway coregulators are also associated with resistance to AR-targeted therapies in CRPC.

The potential mechanisms of canonical AR signaling that lead to CRPC can be categorized into three parts. 1) common alterations in AR, which can lead to AR increase its sensitivity to very low levels of androgens or constitutive activation of AR signaling; 2) AR activation by androgens converted from adrenalandrogens or synthesized intratumorally via the *de novo* route; 3) alterations in cofactors of the AR pathway (Figure 1 **)**.

##### Common Alterations in *AR*

One possible mechanism by which the prostate cancer becomes resistant to androgen deprivation therapy is alterations in *AR*, including *AR* amplification or overexpression, *AR* mutations and truncated *AR* lacking ligand-binding domains (*AR*ΔLBD). Thus, these changes in AR increase its sensitivity to very low levels of androgens or lead to constitutive activation of AR signaling. Strictly speaking, this mechanism of prostate cancers is not androgen-independent and the responses still depend on AR and androgen.

###### *AR* Amplification or Overexpression

Despite low circulating androgens in the CRPC patients, one potential mechanism that would allow tumor cell proliferation is by promoting the expression of the AR itself, which increases ligand-occupied receptor content (Feldman and Feldman, 2001). Plenty of studies have shown that approximately 50% of tumors that become castration resistant after ADT have amplified the AR gene, the most frequent genetic alteration reported for CRPC tumors, whereas none of the untreated primary tumors before androgen ablation had an AR gene amplification (Robinson et al., 2015; Djusberg et al., 2017).

Numerous studies provide the simplest explanation of how increased androgen receptor expression leads to resistance to anti-androgen therapy. According to one study, a three-to-five-fold increase in receptor levels could compensate for low ligand levels and restore androgen receptor signaling in xenotransplantation models. (Chen et al., 2004). Although tumors with AR amplification have increased levels of AR, the signal for cell proliferation presumably continues to require androgen (Visakorpi et al., 1995). Maybe this can explain why tumors with castration resistance have increased sensitivity to androgens in a low androgen environment.

###### *AR* Mutations

In CRPC, the frequency of *AR* mutations in pre-treated tumors is 5–30% (Grasso et al., 2012; Robinson et al., 2015; Kumar et al., 2016). Most mutations identified in CRPC were located in the AR-LBD. These alterations could facilitate AR signaling in CRPC by offering: 1) ligand facilitation, thereby inducing AR activation even in the presence of low or absent levels of androgens and 2) agonist properties to AR antagonists (Coutinho et al., 2016). In addition, mutations can also occur in the AR-NTD that account for about a third of all mutations described in AR. And mutations can usually cause alterations that contribute to AR transactivation, such as facilitated recruitment of co-factors and other components of the transcriptional machinery, promoted N/C interaction, increased response to DHT activation and enhanced protein stability and nuclear retention (Network C. G. A., 2015; Coutinho et al., 2016).

###### Truncated *AR* Lacking Ligand-Binding Domains (*AR*ΔLBD)

Latest RNA sequencing data from big data sets, strongly suggests that constitutively active ARΔLBD may play a role in 40–50% of patients with CRPC (Robinson et al., 2015). Compared with hormone naïve PCa, ARΔLBDs are frequently upregulated in CRPC, and may serve as an adaptive response to therapies targeting the androgen/AR-signaling axis (Guo et al., 2009; Li et al., 2013). The recent genomic data on unique exon junctions reveals that at least 12 distinct *AR-V* mRNA species are detectable in primary PCa and 23 in CRPC (Abeshouse et al., 2015). However, among these variants, AR-V3/AR-V7 appears to be one of the most abundantly and ubiquitously expressed isoforms in our screening of a panel of human prostate cancer cell lines and tissues (Guo et al., 2009; Schweizer and Plymate, 2016). In addition, nonsense mutations leading to premature chain termination (Q641X, formerly Q640X) as well as enzymatic cleavage (tr-AR) were also shown to induce AR△LBDs (Haile and Sadar, 2011). F. Zengerling et al. reported that inhibition of IGF-1R resulted in a down-regulation of AR, Q641X and AR-V7 signaling in PCa cells (Zengerling et al., 2016), which suggests that IGF-1/IGF-1R axis is a modulator of the AR△LBD signaling, providing a rationale by targeting growth factor receptor for CRPC treatment.

##### AR Activation by DHT Synthesized Intratumorally via the *de novo* Route or Converted From Adrenal Androgens

There are two possible ways for the initial substrates to convert to intratumoral DHT in CRPC. The first way is that androgens are synthesized intratumorally via the *de novo* route and the second is that androgens are converted from adrenal androgens.

###### DHT Intratumorally via the *de novo* Route

The use of cholesterol for *de novo* steroidogenesis requires the components of the steroidogenic machinery present in the adrenals and gonads, including steroidogenic acute regulatory proteins, CYP11A1 and CYP17A1 (Miller and Auchus, 2011), which may play important roles in prostate cancer (Locke et al., 2008). Comparisons between primary prostate cancer and CRPC demonstrate that the transcription levels of these proteins are upregulated in CRPC (Montgomery et al., 2008) and CYP17A1 protein is detectable in a subset of metastatic CRPC cases (Efstathiou et al., 2012). In contrast to steroidogenesis in the adrenals and gonads, CRPC expresses steroid-5α-reductase (SRD5A1) with obvious 5α-reductase activity (Chang et al., 2011). One of the functions of robust SRD5A enzyme activity is that any *de novo* steroidogenesis would likely occur through the back door pathway that bypasses the requirement for T and involves 5-reduction of a 21-carbon steroid (progesterone or 17α-hydroxyprogesterone) instead of a 19-carbon androgen (Shaw et al., 2000; Auchus, 2004). Although this biochemical pathway may be engaged in CRPC, the relatively lengthy eight enzymatic steps required for conversion from cholesterol to DHT, the abundance of adrenal precursors present in serum, and the much closer pathway proximity of adrenal precursors to DHT, together suggest that adrenal precursors serve as the major substrate pool.

###### DHT Converted From Adrenal Androgens

There are two possible pathways from adrenal precursor steroids to DHT (Luu-The et al., 2008; Chang and Sharifi, 2012). The canonical adrenal pathway is the route that results in T synthesis as the penultimate metabolite, which undergoes 5-reduction to DHT (Scher and Sawyers, 2005; Ryan and Tindall, 2011; Hofland et al., 2012; Stein et al., 2012). This pathway is probably favored in the field because of the general notion that T must be the precursor to DHT and T is frequently detectable at concentrations greater than DHT in CRPC, as occurs with gonadal androgen physiology (Titus et al., 2005; Montgomery et al., 2008). In this pathway, DHEA is converted by 3β-hydroxysteroid dehydrogenase (3βHSD) to androstenedione (AD), which is then 17-keto reduced by aldo-keto reductase 1C3 (AKR1C3) or 17βHSD3 to T, the immediate precursor to DHT. The second possible pathway is that AD, like T, a 3-keto, △4-steroid, is also a potential substrate for SRD5A (Tomkins, 1957). AD is reduced to 5α-androstanedione (5α-dione), which then becomes the immediate precursor to DHT. The 5α-dione pathway is the major pathway for the synthesis of DHT in CRPC (Chang et al., 2011).

##### Alterations in Cofactors of the AR Pathway

Resistance to AR-targeted therapies in CRPC was also associated with genomic and transcriptomic alterations in coregulators of the AR pathway. Expression of 50 of the ∼200 AR-associated coregulators is aberrant in clinical CRPC specimens (Liu et al., 2017). For example, a higher frequency of mutations in *FOXA1*, the gene encoding a pioneer factor that facilitates AR chromatin binding and transcriptional activation, was found in CRPC (12%) than in primary prostate cancer (4%) (Watson et al., 2013; Abeshouse et al., 2015). In addition, as one class of coregulators, steroid receptor coactivators (SRC-1, SRC-2 and SRC-3) play a key role in facilitating aberrant AR signaling in CRPC e. There have been studies reported that all 3 SRCs is elevated in CRPC (Taylor et al., 2010; Grasso et al., 2012; Abeshouse et al., 2015; Beltran et al., 2016; Bernemann et al., 2016). GATA2, another AR pioneer factor in the AR signaling axis, is aberrant expressed in CRPC and associated with poor outcome (He et al., 2014; Yan et al., 2014). Gupta et al. detected genomic copy number changes of circulating tumor cells from 16 patients with CRPC resistant to abiraterone or enzalutamide and revealed that multiple genes encoding AR coregulators had copy number alterations, including copy number gains BRD4 (43.75%) (Gupta et al., 2017). Moreover, changes of AR corepressors also play a key role in CRPC. For example, loss of activity of the key nuclear receptor corepressors NCOR1 and NCOR2, is prevalent in CRPC due to mutation and/or deletion (Grasso et al., 2012; Abeshouse et al., 2015; Kumar et al., 2016).

#### Non-nuclear AR Signaling

**FIGURE 2:**
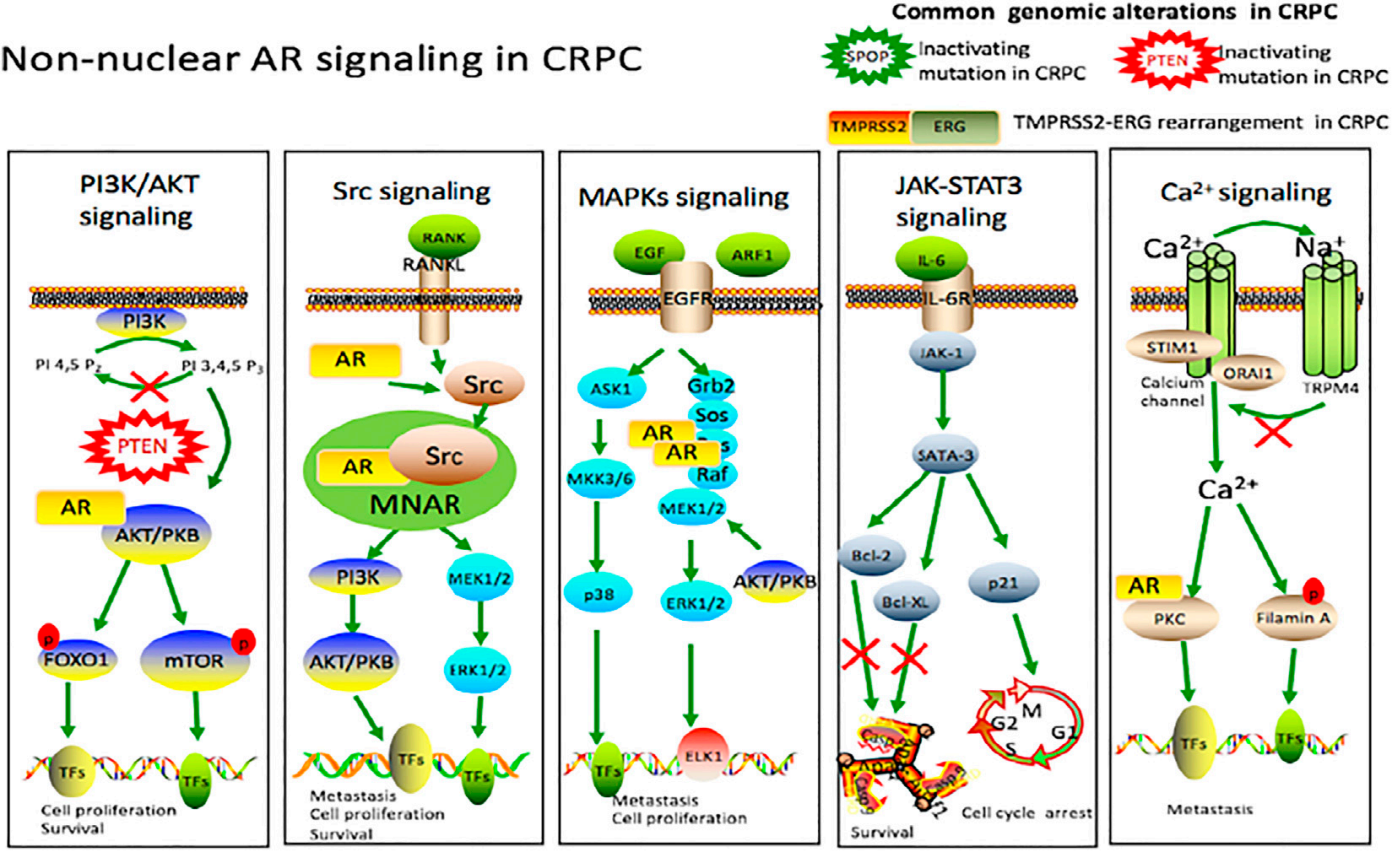
Non-nuclear AR signaling in castration-resistant prostate cancer. Cytokines, interleukins and the growth factors secreted by the prostate cancer cells activate various signaling cascades like PI3K/AKT, Src, MAPKs and JAK/STAT3 pathways involved in castration-resistant prostate cancer, leading to cell proliferation, survival and tumor metastasis. Intracellular Ca^2+^ centration can be modulated through Calcium channel. This increase in intracellular Ca^2+^ can lead to activation of PKC and filamin A, ultimately influencing gene transcription through phosphorylation. TRPM4 is also activated by a rise in intracellular Ca^2+^ in prostate cancer cells. Upon activation, a Na^+^ influx via TRPM4 depolarizes the membrane potential, which decreases the driving force for Ca^2+^, and thus contributes to migration of androgen-insensitive prostate cancer cells. There are other genomic alterations in castration-resistant prostate cancer, including *PTEN* mutation, *SPOP* mutation and *TMPRSS2-ERG* rearrangement.

Trafficking from the nucleus into the cytoplasm, AR may have unexpected consequences because AR has known functions in the cytoplasm, which is called non-genomic signaling (Foradori et al., 2008). One of the main characteristics of non-nuclear signaling is its rapidity with which it occurs. When steroid receptors stay in the cytosol, they can undergo several protein–protein interactions within seconds to minutes after steroid stimulation, which activates a variety of signaling pathways to promote the development of CRPC (Figure 2).

##### PI3K/AKT Signaling Pathway

The PI3K/AKT pathway is one of the most frequently activated signal transduction pathways in human cancer, including prostate cancer(Hoxhaj and Manning, 2020; Park et al., 2018; Braglia et al., 2020). Alterations of the PI3K/AKT pathway, including altered expression, mutation, and copy number alterations, have been reported in 42% of primary prostate tumors and 100% of metastatic tumors (Taylor et al., 2010). Androgens induce the accumulation of TORC2 complex, rapamycin insensitive chaperone of mTOR and stress activated protein kinase interacting protein 1 in the nucleus, thus stimulating TORC2 to activate Akt (Fang et al., 2012). Activated AKT can stimulate many downstream functions via its kinase activity, including glycogen synthase kinase 3 (GSK3), tuberous sclerosis complex (TSC), FOXO transcription factors, NF-kappa-B and Bcl-2 family members BAD, which regulate a range of cellular processes (Liu et al., 2009; Courtney et al., 2010). It is estimated that genomic phosphatase and tensin homolog gene (PTEN) alterations, which is a negative inhibitor of PI3K/AKT pathway, occur in 9–45% of high-grade prostate intra-epithelial neoplasia (HG-PIN), 20–60% of localized prostate cancer, and up to 100% of cases of metastatic prostate cancer (Taylor et al., 2010; Jia et al., 2013).

##### Src Signaling Pathway

Preclinical studies have confirmed that non receptor tyrosine kinase c-Src and Src family kinase (SFK) regulate a complex signal network, driving the development of castration-resistance prostate cancer and bone metastasis. (Cai et al., 2011). After the establishment of bone metastasis, prostate cancer cells destroy the balance of osteoclasts and osteoblasts by secreting a variety of molecules, such as growth factors and cytokines that disrupt the normal process of bone maintenance and reconstruction (Yang et al., 2001; Mundy, 2002). The balance is in favor of osteoblastogenesis, which explains the usual condensing aspect of PCa-derived bone metastases. Src activity specifically affects ruffled borders of osteoclasts (essential for bone resorption), through dynamic regulating the interactions of actin cytoskeleton and formation of podosomes (Horne et al., 2005; Destaing et al., 2011). Src and other SFKs also play important roles in the antiapoptotic signal transduction of RANKL and other tumor necrosis factor family members in osteoclasts (Xing et al., 2001). One essential role for Src in osteoblasts has also been demonstrated that reduction of Src expression decreases osteoblast(responsible for bone formation) proliferation and increases differentiation (Marilena et al., 2000).

Recently, a large number of studies have shown that the activation of SRC is an important mediator of AR signaling. (Asim et al., 2008). AR can form a tertiary complex with the scaffold protein modulator of non-genomic actions of the estrogen receptor (MNAR/PELP1) and Src (Unni et al., 2004). Initially, Src is inactive within this complex. However, when AR binds to Src, this results in the activation of Src in this complex (AR/MNAR/Src) and the subsequent activation of a downstream effector, MEK (Unni et al., 2004). Subsequent studies have shown that AR-induced Src activation can promote cell proliferation through cell cycle progression from G1 phase to S phase (Migliaccio et al., 2002).

##### MAPKs Signaling Pathway

The MAPKs signaling cascade play important roles in regulating diverse biological functions including cell proliferation, motility and survival, which are essential to prostate carcinogenesis (Rossomando et al., 1989; Armenia et al., 2018; Abida et al., 2019). Studies of DHT-responsiveness in prostate cancer cells show that DHT treatment induces phosphorylation of ERK-1/2 within 1–2 min and peak levels of phosphorylation within 5–10 min (Liao et al., 2013). Activated ERK-1/2 then translocate to the nucleus and directly interact with and phosphorylate transcription factors (TFs), such as nuclear ETS domain-containing Elk1 (Marais et al., 1993; Gille et al., 1995; Yang et al., 1998). Elk1 transcriptionally regulates immediate early genes (IEGs) such as c-fos (Gille et al., 1995; Unni et al., 2004), and regulates the expression of several genes related to cell proliferation (Marais et al., 1993; Unni et al., 2004). In addition, recent studies showed that other molecules induce prostate cancer via MAPK signaling. Jason et al. reported that ADP-ribosylation factor 1 (ARF1), a crucial regulator in vesicle-mediated membrane trafficking and involved in the activation of signaling molecules, promotes the occurrence of prostate cancer via targeting oncogenic MAPK signaling (Davis et al., 2016). Gonzalo et al. reported that epidermal growth factor (EGF) could stimulate G0/G1-S transition via p38 MAPK to overcome the growth restriction of androgen deprivation in prostate cancer cells (Rodriguez-Berriguete et al., 2016).

##### JAK-STAT3 Signaling Pathway

Janus kinases (JAK) signal transducers and activator of transcription (STAT) pathway play an important role in differentiation, hematopoiesis, immune function and cell growth (Bolli et al., 2003). Recently, accumulating evidence indicated that IL-6 is indispensable for activation of JAK/STAT pathway, which is involved in the oncogenesis of prostate cancer (Liu X. et al., 2012). Compared with men with normal prostates, benign prostatic hyperplasia, prostatitis and localized disease, approximately 50% of patients with advanced prostate cancer have increased levels of serum IL-6 (Twillie et al., 1995). Upon the binding of IL-6 to the IL-6 receptor, JAK-1 and STAT-3 become activated in sequence (C Schindler and Jr, 1995). L Tam et al. reported that cytoplasmic expression of IL-6 receptor and pSTAT3 Tyr705 are associated with the shortened biochemical recurrence time and death time from hormone relapse, respectively. Therefore, it is reasonable to target this pathway in hormone-refractory prostate cancer treatments (Tam et al., 2007).

##### Ca^2+^ Signaling Pathway

Ca^2+^signaling is also involved in prostate cancer progression (Figiel et al., 2019; Chalmers and Monteith et al., 2018). Increased calcium intake from dairy products has been considered as a risk factor for prostate cancer (Foradori et al., 2007; Flourakis and Prevarskaya, 2009). As a primary signaling molecule, extracellular Ca^2+^ works through the Ca^2+^-sensing receptor (CaR, a G protein coupled receptor) which directly regulates cell signal transduction and the Ca^2+^ channels (Vaz et al., 2015). Depletion of intracellular Ca^2+^ stores serves as a signal for the activation of Ca^2+^ influx across the plasma membrane. The proteins STIM1 and ORAI1 were identified as the key components of store-operated Ca^2+^ entry (SOCE). When Ca^2+^ is released from intracellular Ca^2+^ pool, Ca^2+^ dissociates from a luminal EF hand motif of STIM1. As a consequence, STIM1 proteins aggregate and recruit Orai1 Ca^2+^channels, which then mediate SOCE (Kilch et al., 2016).

Recently, Huang et al. found that Ca^2+^ via CaR-mediated signaling induces filamin A cleavage, which is an actin-binding protein, and promotes the migration of AR-deficient and highly metastatic prostate cancer cells (Huang et al., 2016). In one additional study, Christian demonstrated that transient receptor potential melastatin 4 channel (TRPM4) is activated by a rise in intracellular Ca^2+^ in prostate cancer cells. Upon activation, a Na^+^ influx via TRPM4 depolarizes the membrane potential, which reduces the driving force for Ca^2+^ and limits SOCE, and thus promotes migration of androgen-insensitive prostate cancer cells (Christian et al., 2015).

##### Other Genomic Alterations in Castration-Resistant Prostate Cancer

Prostate cancer is characterized by a high genetic heterogeneity due to genomic alterations and instabilities associated with diverse PCa risk factors (Squire et al., 2011; Yap et al., 2016; Ciccarese et al., 2017; Rodrigues et al., 2017), which was evidenced by extensive genomic profiling analysis conducted on primary tumors (Network T. C. G. A., 2015) and on metastatic samples (Dan et al., 2015).

Speckle-type POZ protein (SPOP) is the substrate-binding subunit of a cullin-3 (CUL3)-based E3 ubiquitin ligase complex, which mediates the ubiquitylation and degradation of many target proteins. SPOP binds to the substrates through its N-terminal meprin and traf homology (MATH) domain, whereas it interacts with cullin-3 via BTB domain on its C terminal (Pintard et al., 2003; Xu et al., 2003; Zhuang et al., 2009). Recent cancer whole-genome and exome sequencing studies have shown that *SPOP* is the most frequently mutated gene (in up to 15% of cases) in primary prostate cancer (Barbieri et al., 2012; Network T. C. G. A., 2015). Interestingly, all *SPOP* somatic mutations identified in prostate cancer are clustered in its substrate binding MATH domain, thus having a dominant-negative effect on substrate binding and degradation (Theurillat et al., 2014). Recent studies have unanimously reported that SPOP interacts with bromodomain and extraterminal (BET) proteins that largely act as transcriptional coactivators and play vital roles in cell cycle, apoptosis, migration and invasion in physiological conditions. In addition, SPOP also promotes the ubiquitylation and proteasomal degradation of bromodomain-containing protein 2 (BRD2), BRD3 and BRD4, (Dai et al., 2017; Janouskova et al., 2017; Zhang et al., 2017). Pathologically, BET proteins are frequently overexpressed and are clinically linked to various types of human cancer (French et al., 2003; Crawford et al., 2008; Belkina and Denis, 2012). Recently, Janouskova *et al.* reported prostate cancer–associated *SPOP* mutants impaired its binding to BET proteins, leading to the reduced proteasomal degradation and accumulation of these proteins in prostate cancer cell lines and patient specimens, which subsequently causes resistance to BET inhibitors (Janouskova et al., 2017). Similar study has also demonstrated that *SPOP*-mutated prostate cancer cell lines and patient-derived organoids were intrinsically resistant to BET inhibitor-induced growth arrest and apoptosis (Dai et al., 2017). Furthermore, Dai et al. provided that stabilization of BRD4 may be a molecular mechanism for resistance to BET inhibitors in patients with prostate cancer bearing *SPOP* mutations (Dai et al., 2017). Taken together, these findings offer mechanistic insights into how *SPOP* mutations influence prostate cancer.

The *TMPRSS2*-*ERG* fusion gene arising from genetic rearrangement (fusion of encoding transmembrane protease serine 2, *TMPRSS2* gene, and *EST*-related gene, *ERG*) has also been a central focus in prostate cancer, which leads to aberrant expression of the *ETS* transcription factor *ERG* (Tomlins et al., 2005; Kandoth, 2013). *TMPRSS2-ERG* is the most common gene rearrangement in prostate cancer and is present in approximately 50% of prostate cancer tissues in Western countries (Cary and Cooperberg, 2013). Previous studies indicated that *ERG* overexpression was driven by hijacking of androgen-responsive elements within the *TMPRSS2* promoter (Tomlins et al., 2005; Wang et al., 2007; Thangapazham et al., 2014). However, Kron et al. found that the molecular process is more complex. Their study indicated that the frequent deletion allows a cluster of regulatory elements (CORE) in the *TMPRSS2* promoter to expand into the rearranged ERG allele. This expanded CORE contains some CREs within the *ERG* locus that can promote *ERG* overexpression. Studies also revealed that overexpressed *ERG* co-opts prostate-specific master regulatory transcription factors, including AR, HOXB13 and FOXA1, in a process facilitated by their physical interaction with ERG and actives NOTCH signaling in primary prostate cancer (Kron et al., 2017). *ERG* overexpression is now an instrumental indicator in the diagnosis of prostate cancer. In addition, Graff et al. recently found that obesity and height might be correlated with the development of *TMPRSS2-ERG*-positive prostate cancer (Graff et al., 2018). Collectively, the functions and mechanisms of *TMPRSS2-ERG* increase the opportunities for finding new therapeutic targets for prostate cancer(Wang et al., 2017; Kong et al., 2020).

## Natural Compounds That Exert Anti-Prostate Effects

Natural compounds that have been found to inhibit prostate cancer cells proliferation/tumor growth, promote prostate cancer cells apoptosis, or modulate specific signaling pathways involved in prostate cancer *in vivo* and *in vitro* are categorized and presented according to their source of isolation (marine organisms, microorganisms and plants) and the structural scaffolds. Besides the effects on prostate cancer cells growth or apoptosis, special emphasis was given to the mechanism of action of a compound interfering specific signaling pathways involved in prostate cancer.

### Natural Compounds Obtained From Marine Organisms or Microorganisms

**FIGURE 3:**
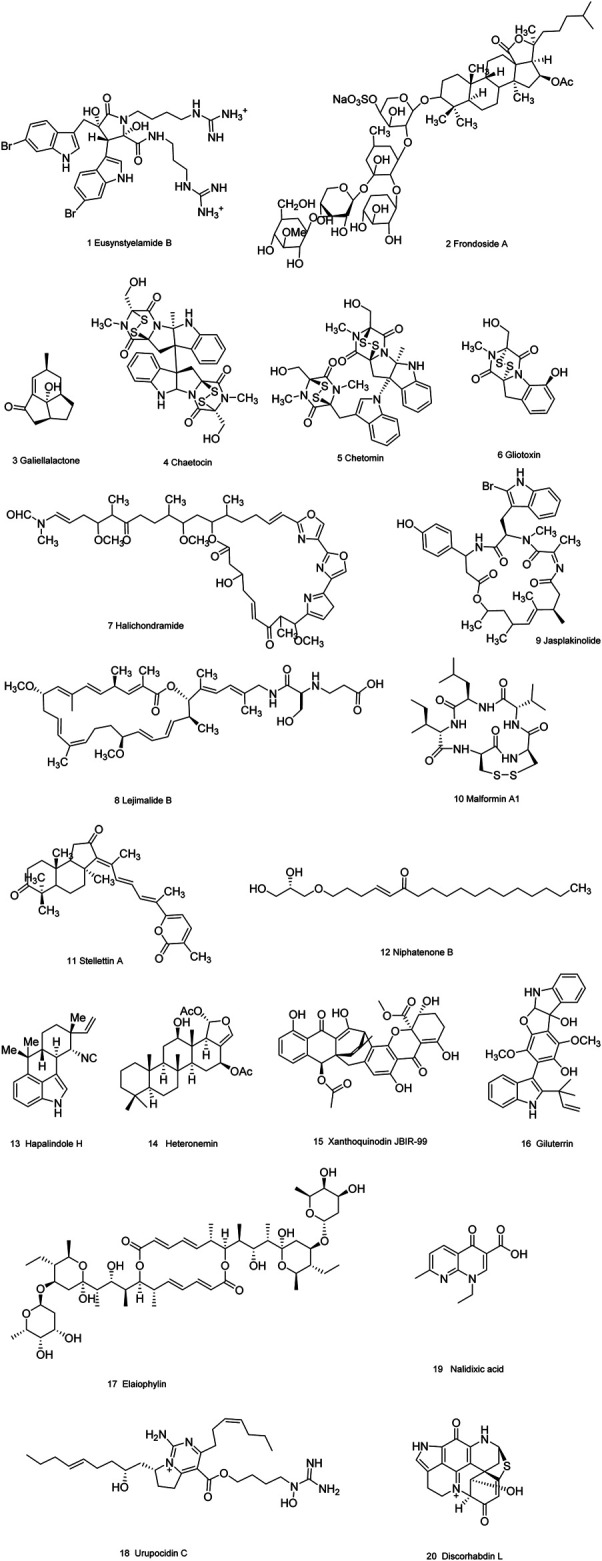
Natural compounds obtained from marine organisms or microorganisms with anti-prostate cancer activities.

As is well known, marine organisms or microorganisms possess the capacity to produce a large amount of diverse secondary metabolites with unique structural features and biological properties. Thus, marine and microbial organisms represent interesting and important sources of single molecules with promising skeletons and significant anti-prostate cancer activity. Up to now, a total of 24 natural compounds (Figures 3) isolated from marine organisms have been found to exhibit significant anti-prostate cancer activity either *in vivo* or *in vitro*. Detailed information about the compounds origin, activity and mechanism of action is listed in Table 1. Most of them exhibit antiproliferative, apoptosis inducing or metastasis inhibitory activities, with various acting mechanisms such as induction of autophagy, inhibition of AR activation, PI-3K/AKT/mTOR or JAK/STAT signaling pathways (Senderowicz et al., 1995; Liu et al., 2006; Wang WL. et al., 2008; Hellsten et al., 2008; Gantar et al., 2012; Meimetis et al., 2012; Shin et al., 2013; Liberio et al., 2015; Liu et al., 2016). Especially, frondoside A not only caused cell type specific cell cycle arrest and induction of caspase-dependent or -independent apoptosis *in vivo* but also significantly inhibited the cell growth of PC-3 and DU145 with a notable reduction of lung metastasis and decrease of circulating tumor cells in the peripheral blood (Dyshlovoy et al., 2016). In addition, gliotoxin, chaetocin and chetomin exhibited antiangiogenic effects *in vitro* and attenuated tumor growth mainly by disrupting the HIF-1α/p300 complex, which makes them attractive molecules for the design of future chemotherapeutic agents (Cook et al., 2009).

**TABLE 1:** Natural compounds obtained from marine organisms or microorganisms with anti-prostate cancer activities.

Natural compound	Sort	Name of microorganisms	Cell type	Observation	Activity	Mechanism of action	Refs
C-phycocyanin	Proteins	*Limnothirix* sp.	LNCaP	*In vitro*	Induction of apoptosis	Increase of radical oxygen species (ROS) generation; increase of caspase-9 and caspase-3 activities.	Gantar et al. (2012)
Eusynstyelamide B(1)	Alkaloids	*Didemnum candidum*	LNCaP	*In vitro*	Antiproliferation	Induction of G2 cell cycle arrest; increase of CHK2 phosphorylation; upregulation of p21CIP1/WAF1; decrease of CDC2 expression.	Liberio et al. (2015)
Frondoside A(2)	Triterpene glycosides	*Cucumaria okhotensis*	DU145 LNCaP PC3 22Rv1 VCaP	*In vivo*	Antiproliferation	Induction of G2/M cell cycle arrest; upregulation of Bax, Bad, PTEN, cleavage of PARP and caspase-3; downregulation of anti-apoptotic proteins (survivin and Bcl-2); inhibition of pro-survival autophagy by upregulation of phospho-mTOR.	Dyshlovoy et al. (2016)
					Induction of apoptosis		
				*In vitro*	Inhibition of metastasis		
					Inhibition of tumor growth		
Galiellalactone(3)	Ketones	*Galiella ruffle*	DU145 LNCaP PC3	*In vivo*	Induction of apoptosis	Inhibition of Stat3 activity; downregulation of the expressions of Bcl-2, Bcl- xL, c-myc and cyclin D1.	Hellsten et al. (2008)
				*In vitro*	Inhibition of tumor growth		
Chaetocin(4)	Ketones	*Trichoderma virens*	PC3	*In vivo*	Antiangiogenesis	Disruption of the HIF-1α/p300 complex.	Cook et al. (2009)
				*In vitro*	Inhibition of tumor growth		
Chetomin(5)	Ketones	*Trichoderma virens*	PC3	*In vivo*	Antiangiogenesis Inhibition of tumor growth	Disruption of the HIF-1α/p300 complex.	Cook et al. (2009)
				*In vitro*			
Gliotoxin(6)	Ketones	*Trichoderma virens*	PC3	*In vivo*	Antiangiogenesis	Disruption of the HIF-1α/p300 complex.	Cook et al. (2009)
				*In vitro*	Inhibition of tumor growth		
Halichondramide(7)	Trisoxazol-e macrolides	*Chondrosia corticata*	PC3	*In vivo*	Antiproliferation Inhibition of metastasis	The suppression of PRL-3 via downregulation phosphoinositide 3-kinase (PI3K) subunits p85 and p110 the expression; downregulation of matrix metalloproteases (MMPs).	Shin et al. (2013)
Lejimalide B(8)	Macrolide-s	*Eudistoma cf. rigida*	LNCaP	*In vivo*	Antiproliferate Induction of apoptosis	Induction of G0/G1 cell cycle arrest and expression of p21waf1/cip1; downregulation of the expression of cyclin A, E, D survivin, p21B and BNIP3.	Wang et al. (2008b)
			PC3				
Jasplakinolide(9)	Cyclopent-apeptides	*Jaspis johnstoni*	LNCaP PC3	*In vitro*	Antiproliferation	Not investigated.	Senderowicz et al. (1995)
			TSUPrl				
Malformin A1(10)	Cyclopent-apeptides	*Aspergillus niger*	LNCaP PC3	*In vitro*	Antiproliferation Induction of apoptosis and necrosis	Induction of mitochondrial damage and autophagy.	Liu et al. (2016)
Stellettin A(11)	Triterpene-s	*Geodia japonica*	LNCaP	*In vivo*	Induction of oxidative stress and apoptosis	Upregulation of FasL and caspase-3 expression.	Liu et al. (2006)
Niphatenone B(12)	Glycerol ethers	*Niphates digitalis*	LNCaP PC3	*In vitro*	Antiproliferation	Binding with the activation function-1 (AF1) region of the AR N-terminus domain (NTD).	Meimetis et al. (2012)
4H-1,3-dioxin-4-one-2,3,6-trimethyl	Dioxin	*Trichoderma atroviride*	PC3	*In vitro*	Induction of apoptosis	Increase of expression of caspase -3	Ks et al. (2019)
				*In vivo*			
Alternol	unknown	*mutant fungus*	PC-3; 22RV1;BPH1	*in vitro*	Antiproliferation;Induction of apoptosis;	Interaction with multiple Krebs cycle enzymes	Li et al. (2019)
Hapalindole H (13)	indole-alkaloid	*Fischerella muscicola*	PC-3	*in vitro*	Antiproliferation;	Through the intrinsic mitochondrial pathway	Acua et al. (2018)
Heteronemin (14)	sesterterpenoid	*Hyrtios* sp.	LNcap; PC3	*In vitro*	Induction of apoptosis	Oxidative and ER Stress Combined with the Inhibition of Topoisomerase II and Hsp90	Lee et al. (2018)
				*In vivo*			
xanthoquinodin JBIR-99 (15)	Quinolines	*Parengyodontium album* MEXU 30054	PC-3	*in vitro*	Induction of apoptosis	Though intrinsic and extrinsic apoptotic pathways	Anaya-Eugenio et al. (2019b)
Giluterrin (16)	alkaloid	*Aspergillus terreus* P63	PC-3	*in vitro*	Antiproliferation	Not investigated	Gubiani et al. (2019)
Elaiophylin (17)	antibiotic	*Actinomycete streptomyces*	22Rv1； VCaP	*in vitro*	blocking RORg transcriptional regulation activities	Inhibition of the expression of RORg target genes AR and AR variant	Zheng et al. (2020)
				*in vivo*			
Urupocidin C (18)	bicyclic guanidine alkaloid	*Monanchora pulchra*	22Rv1;	*in vitro*	Induction of apoptosis	Though mitochondria targeting	Dyshlovoy et al. (2020)
			LNCaP				
Pseudopterosin H	diterpene glycosides	*Pseudopterogorgia elisabethae*	PC-3	*in vitro*	reducing PC-3 cell viability	Inducing apoptosis and downregulating the production of intracellular reactive oxygen species	Bowers et al. (2021)
Nalidixic acid (19)	quinolone antibiotic	*Streptomyces* sp. (C-7)	PC3	*in vitro*	cytotoxic effect	Not investigated	Arora et al. (2018)
Discorhabdin L (20)	alkaloid	*Latrunculia* sp.		*in vivo*	Inhibition of cell growth	Not investigated	Harris et al. (2018)

### Natural Compounds Isolated From Plants

**FIGURE 4:**
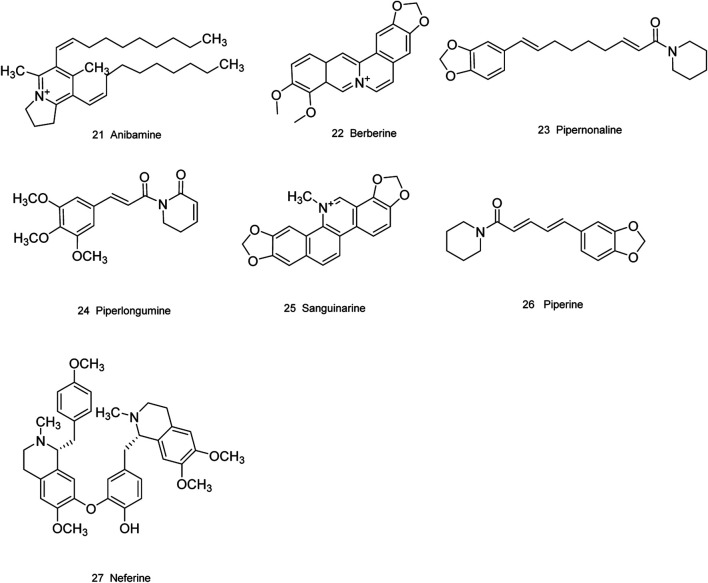
Alkaloids obtained from plants with anti-prostate cancer activities.

**FIGURE 5:**
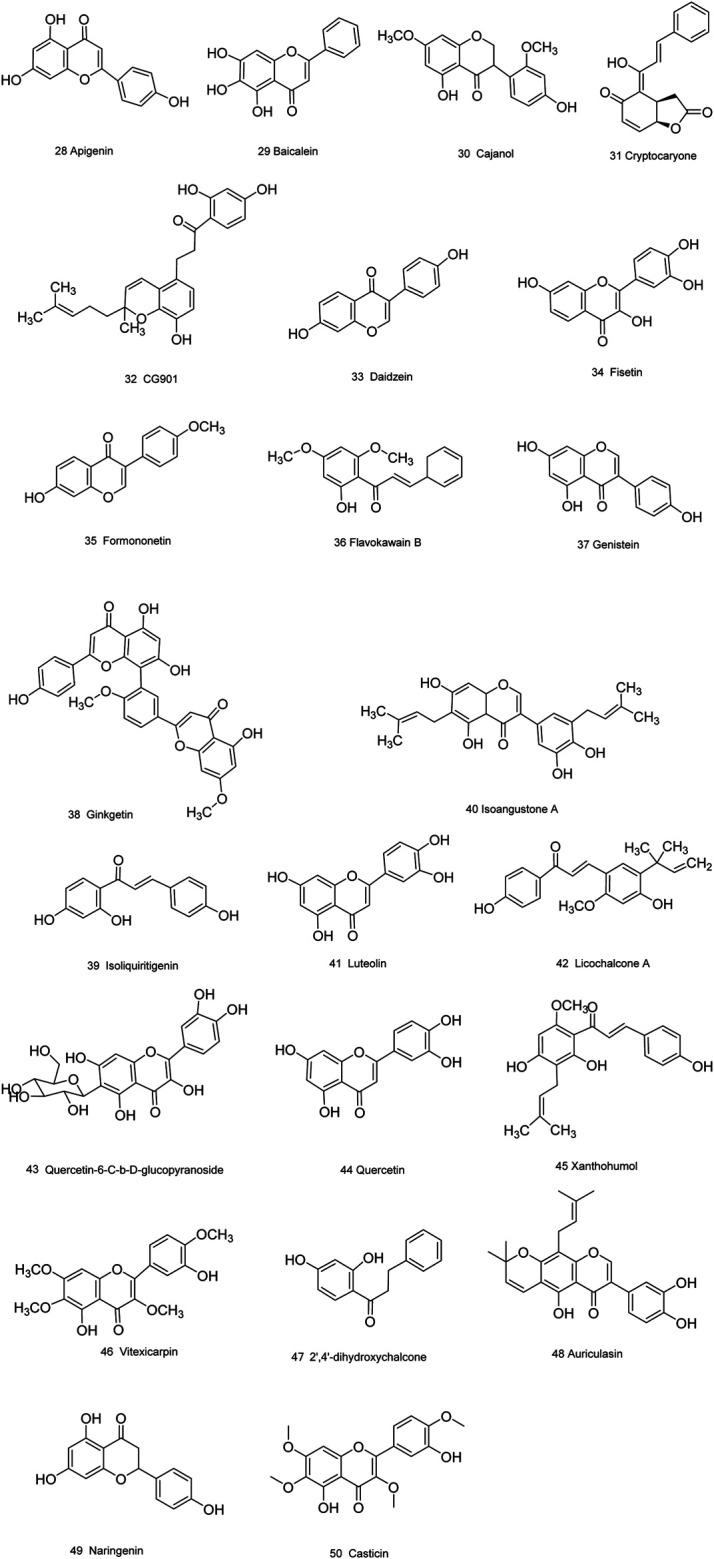
Flavanoids obtained from plants with anti-prostate cancer activities.

**FIGURE 6:**
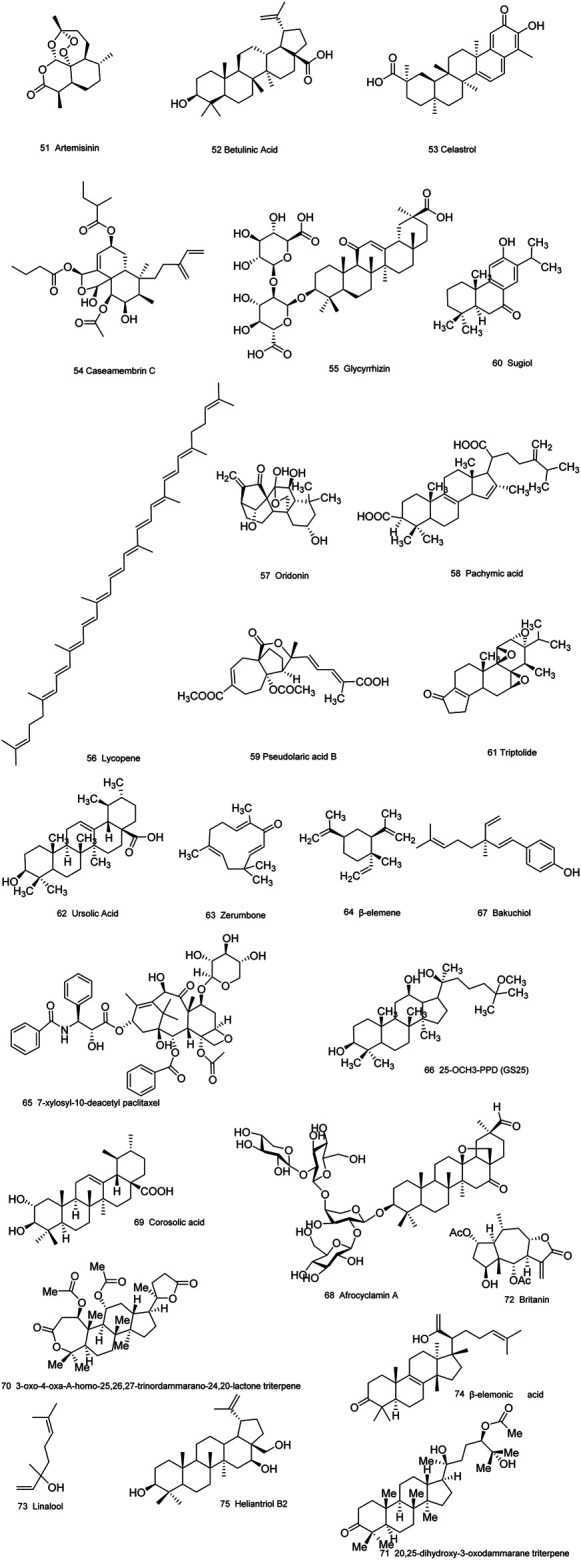
Terpenoids obtained from plants with anti-prostate cancer activities.

**FIGURE 7:**
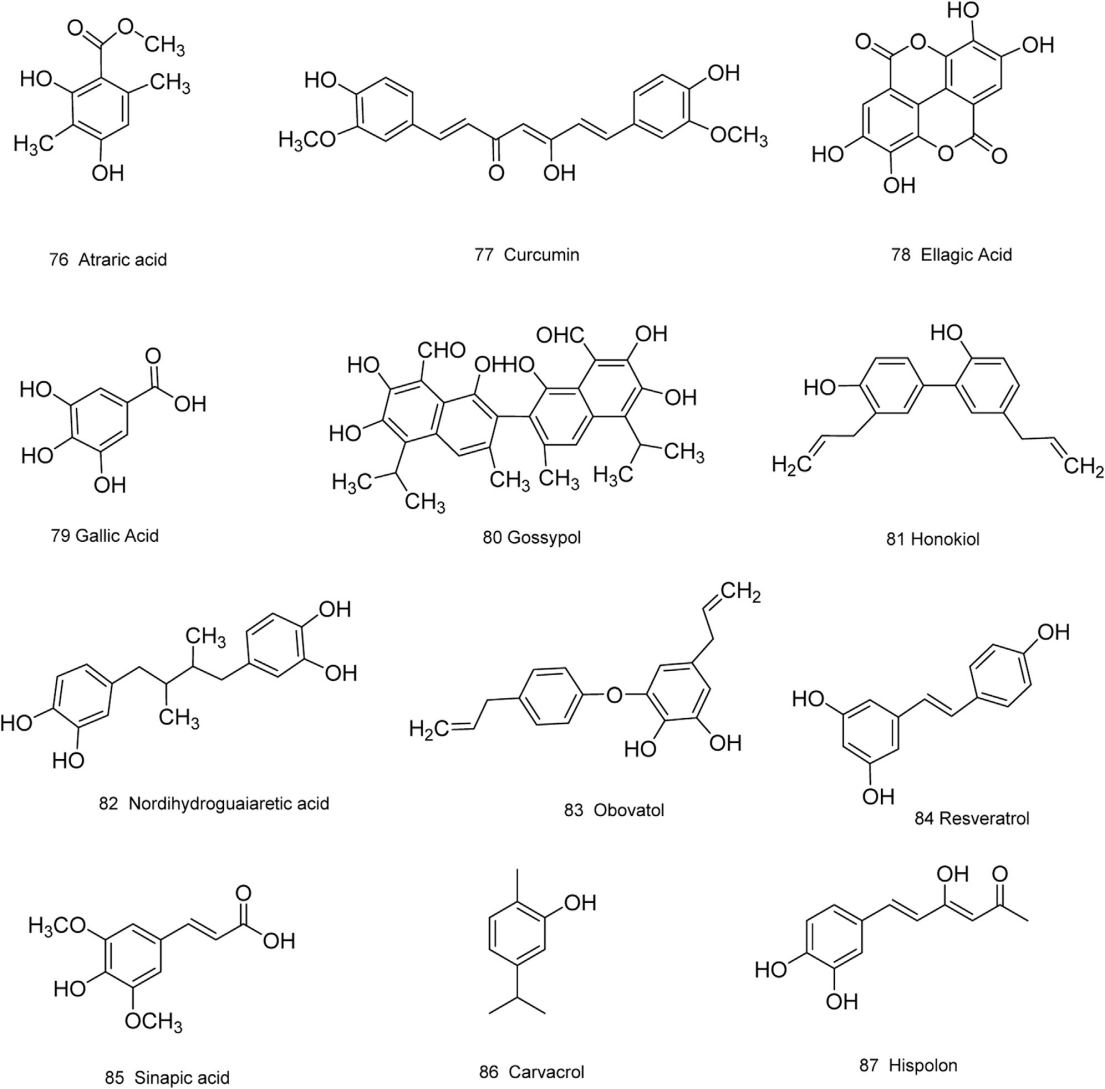
Polyphenols obtained from plants with anti-prostate cancer activities.

**FIGURE 8:**
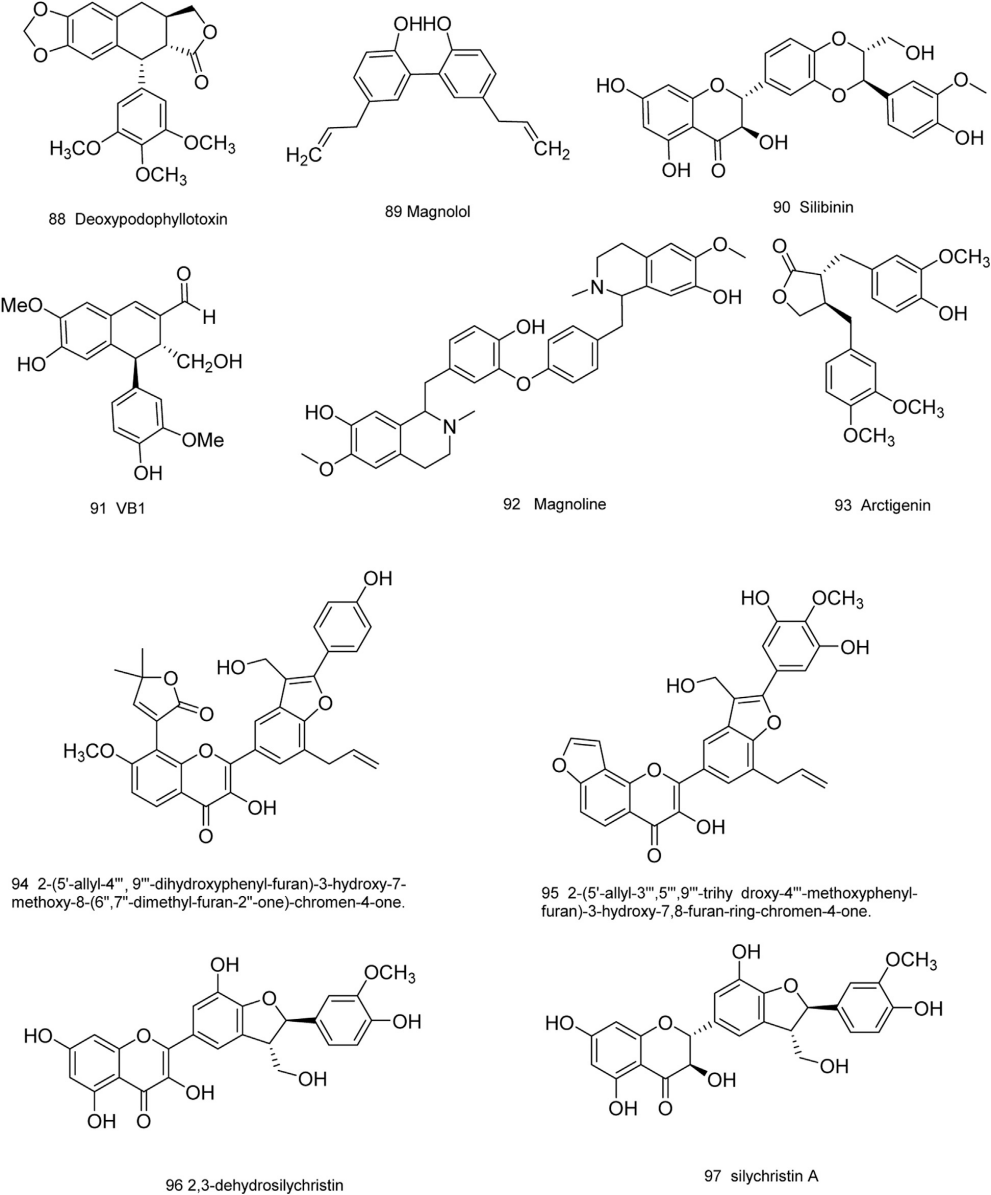
Lignans obtained from plants with anti-prostate cancer activities.

**FIGURE 9:**
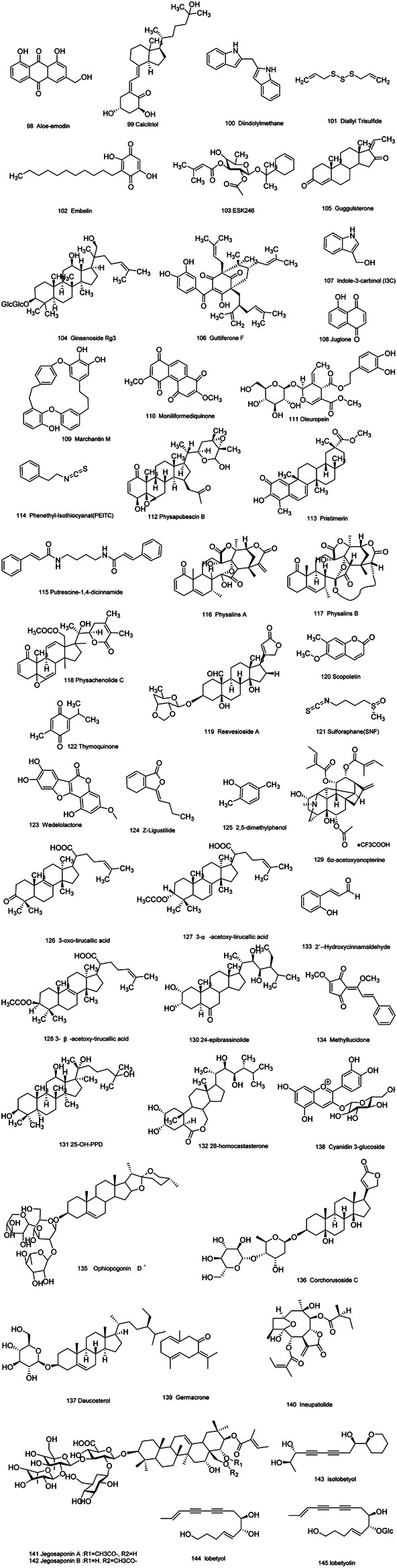
Other compounds obtained from plants with anti-prostate cancer activities.

Medicinal plants have always been a very good source of drugs, which could produce plenty of secondary metabolites with high structural diversity and versatile bioactivities. Many candidates with promising anti-prostate activity have been reported, including 7 alkaloids, 23 flavanoids, 25 terpenoids, 13 polyphenols, 10 lignans and 48 other compounds (Figures 4–9). Almost all these candidates show anti-prostate cancer activities via anti-proliferation, apoptosis induction or metastasis and invasion inhibition, involved in canonical AR signaling and non-AR signaling like caspase cascades, AKT/mTOR pathway, MAPKs pathway, NF-κB pathway, Ca2+ pathway and JAK/STATs pathway. Additionally, there exist other acting mechanisms, for example, anibamine exhibited anti-prostate cancer activity by binding to the chemokine receptor CCR5; fisetin inhibited tumor growth by downregulating the expression of NudC protein, MMP-2 and MMP-9; lycopene showed anti-prostate cancer effects by inhibiting androgen receptor element and signaling of insulin-like growth factor-1 (Afaq et al., 2008; Khan et al., 2008; Bureyko et al., 2009; Wertz, 2009; Zhang et al., 2010b; Chien et al., 2010; Suh et al., 2010; Tang et al., 2011; Holzapfel et al., 2013; Mukhtar et al., 2015). Detailed information is provided in Tables 2–7.

**TABLE 2:** Alkaloids obtained from plants with anti-prostate cancer activities.

Natural compound	Botanical name	Cell type	Observation	Activity	Mechanism of action	Refs
Anibamine(21)	*Aniba panurensis*	DU145 M12	*In vitro*	Antiproliferation	Binding to the chemokine receptor CCR5.	Zhang et al. (2010b)
		PC3		Inhibition of metastasis and invasion		
Berberine(22)	*Coptis chinensis* Franch.	DU145 LNCaP PC3	*In vitro*	Antiproliferation Induction of apoptosis and programmed necrosis	Inhibition of p53-Cyp-D association via decreasing the ROS production; downregulation of HIF-1α and VEGF expression; induction of G1 and G2/M cell cycle arrest by activating ATM-Chk1; induction of caspase-3 and -9 activation; upregulation of bax/bcl-2 expression.	Wang et al. (2012), Zhang et al. (2014)
		PC82 PWR1E	*In vivo*			
Pipernonaline(23)	*Piper longum* L.	DU145 LNCap PC3	*In vitro*	Induction of apoptosis	Induction of sub-G1 and G0/G1 cell cycle arrest through downregulation of CDK2, CDK4, cyclin D1 and cyclin E; upregulation of procaspase-3/PARP cleavage; induction of ROS production and intracellular Ca2+, and mitochondrial membrane depolarization.	Lee et al. (2013b)
Piperlongumine(24)	*Piper longum* L.	LNCaP	*In vitro*	Antiproliferation	Induction of G2/M phase cell cycle arrest; upregulation of bax/bcl-2 expression; activation of caspase-3; downregulation of PARP expression.	Kong et al. (2008)
		PC3				
Sanguinarine(25)	*Argemone mexicana* L.	PC3E RWPE1 TEM4-18	*In vitro*	Cytotoxicity	Inhibition of RGS17 activity.	Bodle et al. (2017)
Piperine (26)	*Piper nigrum* L. *and Piper longum* L.	DU145	*in vitro*	Inhibition of migration progression	downregulating the Akt/mTOR/MMP-9 signaling pathway	Yuan and Ying (2018)
Neferine (27)	*Nelumbo nucifera* Gaertn.	PC3, CD44^+^	*in vitro*	Inhibition of proliferation and migration	through p38 MAPK/JNK activation	Erdogan and Turkekul (2020)
		CSCs； LNCaP				

**TABLE 3:** Flavanoids obtained from plants with anti-prostate cancer activities.

Natural compound	Botanical name	Cell type	Observation	Activity	Mechanism of action	Refs
Apigenin(28)	Distributed in various plants	C4-2B DU145	*In vitro*	Antiproliferation	Binding with IKKα; inhibition of NF-ĸB/p65 activity; inhibition of apoptosis proteins and Ku70-Bax interaction; inhibition of tumor suppressor ER-β degradation; inhibition of class I HDACs expression; inhibition of ABCB1 expression and sensitivity improvement of docetaxel-resistant prostate cancer cells to docetaxel treatment.	Zhu et al. (2013), Salmani et al. (2017)
		PC3	*In vivo*	Anti-invasion		
		22Rv1		Suppression of prostate cancer growth		
Baicalein(29)	*Scutellaria baicalensis* Georgi	LNCaP PC3	*In vitro*	Antiproliferation	Induction of G1 cell cycle arrest; inhibition of androgen receptor (AR) expression.	Haimson et al. (2005)
			*In vivo*	Inhibition of tumor growth		
Cajanol(30)	*Cajanus cajan* (L.) Huth	PC3	*In vitro*	Induction of apoptosis	Induction of G1 and G2/M cell cycle arrest; modulation of the ERa-dependent PI3K pathway and induction of GSK3 and CyclinD1 activation.	Haimson et al. (2005)
Cryptocaryone(**31**)	*Cryptocarya wightiana* Thwaites	PC3	*In vitro*	Antiproliferation	Induction of caspase-8 and 3 activation; upregulation of DR5 surface expression; induction of Fas clustering and the association of downstream signaling molecules, including FADD and procaspase-8; induction of DR4 and DR5 aggregation.	Chen et al. (2010)
				Induction of apoptosis		
CG901(32)	*Artocarpus altilis* (Parkinson) Fosberg	C4-2B DU145	*In vitro*	Antiproliferation	Selective inhibition of prostate cancer cell lines proliferation and mouse xenograft growth by inhibiting the expression of STAT3 target genes.	Jeon et al. (2015a)
		PC3	*In vivo*			
Daidzein(33)	*Glycine max* (L.) Merr.	DU145	*In vitro*	Antiproliferation	Decrease the expression of VEGF and AR genes; induction of G2/M phase in the PC3 cells by downregulating Cyclin B1 and CDK1, and upregulating CDK inhibitors (p21 and p27); upregulation of Fas ligand (FasL) and the expression of proapoptotic Bim; downregulation of the expression of p-FOXO3a and increase of the nuclear stability of FOXO3a.	Labow and Layne, (1972)
		LNCaP PC3	*In vivo*	Induction of apoptosis		
Fisetin(34)	Distributed in various plants	DU145 LNCaP PC3	*In vitro*	Antiproliferation	Downregulation of the expression of NudC protein; downregulation of MMP-2 and MMP-9 expressions.	(Shimoi, 1998; Afaq et al., 2008; Khan et al., 2008; Chien et al., 2010; Suh et al., 2010; Mukhtar et al., 2015)
		22Rv1	*In vivo*	Inhibition of tumor growth		
Formononetin(35)	*Trifolium pratense* L.	PC3	*In vitro*	Antiproliferation	Induction of G0/G1 phase cell cycle arrest; inhibition of the IGF-1/IGF-1R pathway and alteration of the Bax/Bcl-2 ratio; downregulation of expression levels of cyclin D1 and CDK4.	(Huang et al., 2013; Bi, 2014; Li et al., 2014)
		RWPE1	*In vivo*	Induction of apoptosis		
Flavokawain B(36)	*Piper methysticum* G.Forst.	DU145	*In vitro*	Antiproliferation	Selective inhibition on androgen receptor (AR)-negative prostate cancer cell growth; induction of apoptosis with associated increased expressions of proapoptotic proteins: death receptor-5, Bim, Puma and downregulation of XIAP and survivin expressions.	Tang et al. (2010)
		PC3	*In vivo*	Induction of apoptosis		
		LNCaP				
		LAPC4				
Genistein(37)	*Glycine max* (L.) Merr*.*	DU145 PC3 PC-3M	*In vitro*	Inhibition of migration	Prevention of metastasis by directly binding to MEK4 and downregulation of p38 expression; downregulation of expression and activity of MMP-2.	(Li and Sarkar, 2002; Hsu et al., 2010)
			*In vivo*			
Ginkgetin(38)	*Ginkgo biloba* L.	DU145	*In vitro*	Antiproliferation Induction of apoptosis Inhibition of tumor growth	Induction of G0/G1 cell cycle arrest; selective suppression of STAT3 Tyr705 phosphorylation but not through inhibiting upstream tyrosine kinases and tyrosine phosphatase.	Jeon et al. (2015b)
		LNCaP PC3	*In vivo*			
Isoliquiritigenin(39)	Distributed in various plants	D4-2B	*In vitro*	Antiproliferation	Downregulation of the expression of NudC protein; downregulation of MMP-2 and MMP-9 expressions.	Zhang et al. (2010a)
		LNCaP	*In vivo*	Inhibition of tumor growth		
Isoangustone A(40)	*Ginkgo biloba* L.	DU145 PC3	*In vitro*	Antiproliferation	Induction of p27kip1 tumor suppressor gene accumulation by attenuating p27kip1 at Thr 187 phosphorylation; inhibition of CDK2 activation through binding with the CDK2 complex; inhibition of mTOR kinase activity by binding with the mTOR complex.	Lee et al. (2013a)
			*In vivo*	Induction of apoptosis		
				Inhibition of tumor growth		
Luteolin(41)	Distributed in various plants	LNCaP PC3	*In vitro*	Antiproliferation	Inhibition of ANO1 expression; inhibition of the expression and/or function of ARs via regulation of prostate derived Ets transcription factor(PDEF); inhibition of IGF-1/IGF-1R system; decrease of the expression of E-cadherin through MDM.	Seo et al. (2017)
			*In vivo*	Prevention of metastasis		
Licochalcone A(42)	*Glycyrrhiza glabra* L.	LNCaP	*In vitro*	Antiproliferation	Induction of G2/M cell cycle arrest; downregulation of cyclin B1 and cdc2 expression; inhibition of phosphorylation of retinoblastoma (Rb); decrease of expression of transcription factor E2F, cyclin D1, CDKs 4 and 6; increase of expression of cyclin E.	Su et al. (2017)
				Induction of apoptosis		
Quercetin-6-C-b-D-glucopyranoside(43)	*Ulmus wallichiana* Planch.	DU145	*In vitro*	Antiproliferation	Induction of apoptosis via induction of cleaved PARP and Cas-3; induction of G0/G1 cell cycle arrest; inhibition of reactive oxygen generation and AKT/mTOR survival pathway via directly binding with aryl hydrocarbon receptor.	Hamidullah et al. (2015)
		LNCaP PC3		Induction of apoptosis		
		RWPE1				
Quercetin(44)	Distributed in various plants	DU145	*In vitro*	Induction of apoptosis	Increase of expression of c-Jun and its phosphorylation; decrease of expression of HSP72; intervention of BaP toxicity.	Asea et al. (2001), Yuan et al. (2004), Chaudhary et al., (2007), Aalinkeel et al. (2008)
		LNCaP PC3	*In vivo*			
Xanthohumol(45)	*Humulus lupulus* L.	BPH-1 PC3	*In vitro*	Antiproliferation	Induction of S and Sub G1 cell cycle arrest; inhibition of NFkB activity and expression. Upregulation of pro- apoptotic proteins Bax and p53 expression.	Colgate et al. (2007)
				Induction of apoptosis		
Vitexicarpin(46)	*Vitex rotundifolia* L.f.	PC3	*In vitro*	Induction of apoptosis	Induction of G2/M cell cycle arrest.	Meng et al. (2012)
2′,4′-dihydroxychalcone (47)	*Herba oxytropis*	PC3	*In vitro*	Induction of apoptosis	Induction of G1/S cell cycle arrest.	Sheng et al. (2015)
Auriculasin (48)	*Flemingia macrophylla* var. *philippinensis* (Merr. & Rolfe) H.Ohashi	LNCaP-FGC；RWPE-1	*In vitro*	Induction of apoptosis; antiproliferation	Regulation of the PI3K/AKT/mTOR pathway in LNCaP prostate cancer cells;ROS accumulation	Cho et al. (2018)
			*In vivo*			
Naringenin (49)	*citrus fruits and tomatoes*	MAT-LyLu cells	*In vitro*	Inhibition of metastasis	Blocking voltage-gated sodium channels	Gumushan Aktas and Akgun (2018)
Casticin (50)	*Vitex rotundifolia* L.f.,	DU 145	*In vitro*	Inhibition of prostate cancer cell metastasis	Inhibition of the protein levels of AKT, GSK3αβ, Snail, and MMPs (MMP-2, -9, -13, and -7);diminision of the expressions of NF-κB p65, GRB2, SOS-1, MEK,p-ERK1/2, and p-JNK1/2	Lin et al. (2019)

**TABLE 4:** Terpenoids obtained from plants with anti-prostate cancer activities.

Natural compound	Botanical name	Cell type	Observation	Activity	Mechanism of action	Refs
Artemisinin(51)	*Artemisia annua* L.	DU145	*In vitro*	Induction of apoptosis	Increase of synthesis, and cleavage of procaspase-9, cleavage of caspase-3, and PARP-1 degradation.	Nakase et al. (2009)
		PC3				
Betulinic Acid(52)	*Bacopa monnieri* (L.) Wettst.	LNCaP	*In vitro*	Induction of apoptosis	Activation of selective proteasome-dependent degradation of the transcription factors specificity protein 1(Sp1), Sp3, and Sp4; regulation of survivin and VEGF expression; downregulation of NF-kappaB expression.	Chintharlapalli et al. (2007)
			*In vivo*	Inhibition of tumor growth		
Celastrol(53)	*Tripterygium wilfordii* Hook.f.	LNCaP PC3	*In vitro*	Induction of apoptosis	Accumulation of ubiquitinated proteins and three natural proteasome substrates IKB-A, Bax, and p27.	Wolfram et al. (2014)
			*In vivo*			
Caseamembrin C(54)	*Casearia membranacea*	PC3	*In vitro*	Antiproliferation	Downregulation of Bcl-2 and Bcl-xL expression; upregulation of Mcl-1S protein and activation of caspase-9 and caspase-3.	Huang et al. (2004)
				Induction of apoptosis		
Glycyrrhizin(55)	*Glycyrrhiza glabra* L.	DU145 LNCaP	*In vitro*	Antiproliferation	Downregulation of the expression of caspase-3 and caspase-8.	Thirugnanam et al. (2008)
				Induction of apoptosis		
Lycopene(56)	Distributed in various plants	PC3	*In vitro*	Antiproliferation Antimetasis	Inhibition of the androgen receptor element, resulting in decreased PSA velocity; inhibition signaling of insulin-like growth factor-I (IGF-I); decrease of the expression of αvβ3 and αvβ5 integrin.	(Bureyko et al., 2009; Wertz, 2009; Tang et al., 2011; Holzapfel et al., 2013)
			*In vivo*	Induction of apoptosis		
Oridonin(57)	*Isodon rubescens* (Hemsl.) H.Hara	PC3	*In vivo*	Induction of apoptosis	Increase of expression of P21 and the mRNA level of beclin; increase of caspase-3 activity.	(Deepak and Handa, 2000; Xiang et al., 2012; Ming et al., 2016)
			*In vitro*	Inhibition of tumor growth		
				Antiproliferation		
Pachymic acid(58)	*Poria cocos*	DU145 LNCaP	*In vitro*	Antiproliferation	Decrease of prostaglandin synthesis and AKT activity.	Gapter et al. (2005)
				Induction of apoptosis		
Pseudolaric acid B(59)	*Larix kaempferi* (Lamb.) Carrière	DU145	*In vitro*	Antiproliferation	Increase of ROS generation and Bcl-2 degradation.	Zhao et al. (2012)
				Induction of apoptosis		
Sugiol(60)	Salvia prionitis Hance	DU145	*In vitro*	Antiproliferation	Induction of G0/G1 cell cycle arrest; downregulation the expression of STAT3; interaction with TKT.	Jung et al. (2015)
		LNCaP				
		PC3				
Triptolide(61)	*Tripterygium wilfordii* Hook.f.	DU145	*In vitro*	Antiproliferation	Decrease of CDK7-mediated phosphorylation; disruption of the phosphorylation of AR through XPB/CDK7.	Fei et al. (2016)
		LNCaP PC3	*In vivo*	Induction of apoptosis		
		22Rv1				
Ursolic Acid(62)	*Distributed in various plants*	DU145 LNCaP	*In vitro*	Antiproliferation	Upregulation of DR5 activation of JNK; inhibition of NF-κB and STAT3 pathways.	(Deepak and Handa, 2000; Shanmugam et al., 2011; Meng et al., 2015)
			*In vivo*	Induction of apoptosis		
Zerumbone(63)	*Zingiber zerumbet* (L.) Roscoe ex Sm.	DU145	*In vitro*	Antiproliferation	Increase of MPM-2 expression; increase of Bcl-2 and Bcl-xL phosphorylation; induction of Cdk1 activity; induction of Cdc25C downregulation.	Chan et al. (2015)
				Induction of apoptosis		
		HRPC				
		PC3				
β-elemene(64)	Distributed in various of plants	DU145 PC3	*In vitro*	Antiproliferation	Downregulation of Bcl-2 expression; increase of cytochrome c; activation of PARP and caspase-3, -7, -9, and -10.	Li et al. (2010)
				Induction of apoptosis		
7-xylosyl-10-deacetyl paclitaxel(65)	*Taxus wallichiana* Zucc.	PC3	*In vitro*	Antiproliferation	Induction of G2/M cycle arrest; upregulation of pro-apoptotic Bax and Bad protein expressions and downregulation of anti- apoptotic Bcl-2 and Bcl-XL expressions.	Jiang et al. (2008)
				Induction of apoptosis		
25-OCH3-PPD (GS25)(66)	*Panax notoginseng* (Burkill) F.H.Chen	DU145	*In vitro*	Induction of apoptosis	Decrease of MDM2 protein level; increase of the protein levels of the wild-type p53, Bax, cleaved-PARP.	Wang et al. (2008a)
		LNCAP	*In vivo*			
		PC3				
Bakuchiol (67)	*Cullen corylifolium* (L.) Medik.	PC3	*In vitro*	Inhibition of cell proliferation and migration;	Inactivating NF-κB signaling via AR and ERβ	Miao et al. (2017)
Afrocyclamin A (68)	*Androsace umbellata* (Lour.) Merr.	DU145	*In vitro*;	Induction of apoptosis;inhibition of migration and invasion; inhibition of cell growth	Via the PI3k/Akt/mTOR pathway	Sachan et al. (2018)
			*In vivo*			
Corosolic acid (69)	*Eriobotrya japonica* (Thunb.) Lindl*.; Crataegus pinnatifida* Bunge*; Actinidia chinensis* Planch.	PC-3;DU145;22RV1;WPMY-1	*In vitro*	Inhibition of cell growth;Induction of apoptosis	The activation of endoplasmic reticulum (ER) stress-associated two pro-apoptoticsignaling pathways	Ma et al. (2018)
3-oxo-4-oxa-A-homo-25,26,27-trinordammarano-24,20-lactone triterpene (70)	*Cleome khorassanica* Bunge & Bien	DU-145 ； LNCaP	*In vitro*	Cell growth inhibition	Not investigated	Sajjadi et al. (2018)
20,25-dihydroxy-3-oxodammarane triterpene (71)	*Cleome khorassanica* Bunge & Bien	DU-145 ； LNCaP	*In vitro*	Cell growth inhibition	Not investigated	Sajjadi et al. (2018)
Britanin (72)	*Inula linariifolia* Turcz.	PC-3； PC-3-LU； DU-145	*In vitro*;	Inhibition of cell proliferation, migration, and motility	Through PI3K/Akt/NF-κB Signaling Pathways	Zeng et al. (2020)
			*In vivo*			
Linalool (73)	*herbs, spices and fruits*	22Rv1	*In vitro*;	Antiproliferation;Induction of apoptosis;Inhibition of migration invasion	Mitochondria-mediated intrinsic and death-receptor-mediated extrinsic pathways; inhibition of expression of Ki-67 and PCNA in the 22Rv1 xenograft model.	Zhao et al. (2020)
			*In vivo*			
β-elemonic acid (74)	*Boswellia carterii* Birdw	DU145, PC-3 and 22RV1	*In vitro*	Induction of apoptosis	Through the suppression of JAK2/STAT3/MCL-1 and NF-ĸB signal pathways	Bao et al. (2021)
Heliantriol B2 (75)	*Chuquiraga erinacea* subsp. erinacea (Asteraceae)	PC-3;	*In vitro*	Antiproliferation;Induction of apoptosis;Inhibition of migration invasion	Not investigated	Castro et al. (2019)
		LNCaP				

**TABLE 5:** Polyphenols obtained from plants with anti-prostate cancer activities.

Natural compound	Botanical name	Cell type	Observation	Activity	Mechanism of action	Refs
Atraric acid (76)	*Prunus africana* (Hook.f.) Kalkman	LNCaP	*In vitro*	Inhibition of prostate cancer cell growth	Inhibition of AR nuclear translocation.	Schleich et al. (2006)
Curcumin(77)	*Curcuma longa* L.	DU145	*In vitro*	Antiproliferation	Inhibition of the expression of MT1-MMP and MMP2 proteins; inhibition of the DNA-binding ability of NICD.	Yang et al. (2017)
		PC3				
Ellagic Acid(78)	Distributed in various plants	LNCaP	*In vitro*	Antiproliferation Induction of apoptosis	Increase of Bax/Bcl2 ratio and increase caspases 3, caspases 6, caspases 8, and caspases 9 and PARP cleavage; inhibition of mTOR activation and reduction of intracellular levels of β-catenin; downregulation of the expressions of anti-apoptotic proteins, silent information regulator 1 (SIRT1), human antigen R (HuR) and heme oxygenase-1 (HO-1).	Vanella et al. (2013)
Gallic Acid(79)	*Toona sinensis* (Juss.) M.Roem.	DU145 PC3 22Rv1	*In vitro*	Inhibition of migration Induction of apoptosis	Activation of Chk1 and Chk2 and inhibition of Cdc25C and Cdc2 activities; blocking of the p38, JNK, PKC and PI3K/AKT signaling pathways and downregulation of NF-κB protein level; inhibition of MMP2 and MMP 9 gene expression.	(Hastak et al., 2003; Kaur et al., 2009)
			*In vivo*			
Gossypol(80)	*Gossypium hirsutum* L.	DU145 PC3	*In vitro*	Antiproliferation Induction of apoptosis	Downregulation of Bcl-2 and Bcl-xl and the upregulation of Bax; activation of caspase3, caspase8 and caspase9 through the ROS-independent mitochondrial dysfunction pathway and the increase of PARP cleavage; suppression of the expression of AP-1and NF-κB blocked the activation of VEGF receptor 2 kinase.	Huang et al. (2006)
			*In vivo*			
Honokiol(81)	Magnolia officinalis	PC3 LNCaP	*In vitro*	Induction of apoptosis	Activation of Bax and/or Bak; decrease of expression of c-Myc.	Shigemura et al. (2007)
		Myc-CaP	*In vivo*	Inhibition of prostate tumor growth		
Nordihydroguaiaretic acid(82)	*Larrea tridentata* (DC.) Coville	PC3	*In vitro*	Inhibition of migration Antiproliferation	Suppression of NRP1 function.	Li et al. (2016)
			*In vivo*			
Obovatol(83)	*Magnolia obovata* Thunb.	PC3 LNCaP	*In vitro*	Induction of apoptosis	Inhibition of TNF-α and TPA-induced DNA binding activity of NF-κB; translocation inhibition of p65 and p50 into nucleus via decreasing IκB phosphorylation; increase of the apoptotic genes expression: Bax, caspase-3, caspase-9; inhibition of the anti-apoptotic genes expression: Bcl-2, inhibitor of apoptosis protein (IAP-1) and X chromosome IAP (XIAP).	Soyong et al. (2009)
Resveratrol(84)	*Reynoutria japonica* Houtt.	DU145	*In vitro*	Induction of apoptosis	Downregulation of Bcl-2 and Bcl-xL and upregulation of Bax; activation of caspases-3, -8 and -9 and increased PARP cleavage.	Chang et al. (2013)
			*In vivo*			
Procyanidin	*Arachis hypogaea* L.	DU145	*In vitro*	Antiproliferation	Induction of apoptotic cell death and cell cycle arrest at S phase;increase of intracellular ROS level and the decrease of Bcl-2/Bax ratio, and the activation of p53 and caspases-3	Chen et al. (2018)
Sinapic acid (85)	*various vegetables and fruit species*	PC3;	*In vitro*	Antiproliferation;Induction of apoptosis;Inhibition of migration invasion	Increase of the expression of BAX, CASP3, CASP8, CYCS, FAS, TIMP-1 and CDH1,decrease of expression of MMP-9 in PC-3 cells;decrease of in the expressions of CDH2, MMP-2 and MMP-9 in LNCaP cells;increase of caspase-3 activity	Eroğlu et al. (2018)
		LNCaP				
Carvacrol (86)	*Origanum and Thymus*	Human OSCC Tca-8113; SCC-25	*In vitro*	Antiproliferation, inhibition of metastasis and invasion;Induction of apoptosis	Regulating the cell cycle-associated proteins (P21, CCND1 and CDK4) and apoptosis-associated proteins (Cox2, Bcl-2, and Bax); inhibiting P-FAK, and reducing β-catenin, ZEB1, and MMP-2/9 expression	Dai et al. (2016)
Hispolon (87)	*Phellinus linteus*	DU145	*In vitro*	Induction of apoptosis	Via modulation of mitochondrial and STAT3 pathways	Masood et al. (2019)

**TABLE 6:** Lignans obtained from plants with anti-prostate cancer activities.

Natural compound	Botanical name	Cell type	Observation	Activity	Mechanism of action	Refs
Deoxypodophyllotoxin(88)	*Anthriscus sylvestris* (L.) Hoffm.	LNCaP PC3	*In vitro*	Antiproliferation	Accumulation of the reactive oxygen species, intracellular Ca^2+^; increase of mitochondrial membrane potential.	Kim et al. (2013)
				Induction of apoptosis		
Magnolol (89)	*Magnolia officinalis* Rehder & E.H.Wilson	PC3	*in vivo*	Cytotoxicity	Decrease of MMP-2 and MMP-9 expression; decrease of the level of phosphorylated AKT.	Lee et al. (2009)
		PrEC				
Silibinin(90)	*Silybum marianum* (L.) Gaertn.	LNCaP 22Rv1	*In vitro*	Antiproliferation	Activation of acetyl-CoA carboxylase; reduction in hypoxia-induced NADPH oxidase (NOX) activity; increase of the lipid accumulation and NOX activity; downregulation of HIF-1α expression, lipid levels, clonogenicity and NOX activity.	Ting et al. (2016)
			*In vivo*			
VB1(91)	*Vitex negundo* L.	PC3	*In vitro*	Induction of apoptosis	Activation of PARP cleavage.	Zhou et al. (2009)
			*In vivo*			
Magnoline (92)	*Phellodendri amurensis cortex*	22RV1	*In vitro*	Antiproliferation	Disturbance of nutrition metabolism and energy metabolism	Sun et al. (2018)
Arctigenin (93)	*Arctium lappa* L.	PC-3M	*In vitro*	Induction of apoptosis and autophagy	Via PI3K/Akt/mTOR inhibition	Sun et al. (2021)
2-(5′-allyl-4‴,9‴-dihydroxyphenyl-furan)-3-hydroxy-7-methoxy-8-(6″,7″-dimethyl-furan-2″-one)-chromen-4-one. (94)	*Hosta plantaginea* (Lam.) Aschers	LNCaP	*In vitro*	Inhibition of cell growth	Not investigated	Wei et al. (2020)
2-(5′-allyl-3‴,5‴,9‴-trihydroxy-4‴-methoxyphenyl-furan)-3-hydroxy-7,8-furan-ring-chromen-4-one. (95)	*Hosta plantaginea* (Lam.) Aschers	LNCaP	*In vitro*	Inhibition of cell growth	Not investigated	Wei et al. (2020)
2,3-dehydrosilychristin (96)	*Hosta plantaginea* (Lam.) Aschers	LNCaP	*In vitro*	Inhibition of cell growth	Not investigated	Wei et al. (2020)
silychristin A (97)	*Hosta plantaginea* (Lam.) Aschers	LNCaP	*In vitro*	Inhibition of cell growth	Not investigated	Wei et al. (2020)

**TABLE 7:** Other compounds obtained from plants with anti-prostate cancer activities.

Natural compound	Sort	Botanical name	Cell type	Observation	Activity	Mechanism of action	Refs
Aloe-emodin(98)	Anthraquin-ones	*Rheum palmatum* L.	PC3	*In vitro*	Antiproliferation	Binding with mTOR complex2；Inhibition of mTORC2 kinase activity and downstream substrates of mTORC2, AKT and PKCa activity.	Liu et al. (2012a)
				*In vivo*	Suppression of prostate cancer growth		
Calcitriol(99)	Secosteroids	Distributed in various plants	C4-2B	*In vitro*	Antiproliferation	Upregulation of Vitamin D receptor (VDR) expression; induction of BAX expression; increase of cleaved caspase-3 and downregulation of cdk2 expression.	Ben-Eltriki et al. (2016)
			LNCaP		Induction of apoptosis		
Diindolylmethane(100)	Indoles	Distributed in various plants	LNCaP PC3	*In vitro*	Antiproliferation Induction of apoptosis	Induction a [Ca^2+^] rise by evoking phospholipase C-dependent Ca^2+^ release from the endoplasmic reticulum and Ca^2+^ entry via phospholipase A2-sensitive store-operated Ca^2+^ channels; regulation of FOXO3a/β-catenin/GSK-3β signaling; regulation of estrogen metabolism and acting as an antiandrogen, finally leading downregulation of the AR and PSA.	Wang et al. (2016a)
Diallyl Trisulfide(101)	Trisulfides	*Allium sativum* L.	DU145	*In vitro*	Induction of apoptosis	Downregulation of XIAP protein expression.	Kim et al. (2011)
			LNCap PC3	*In vivo*			
Embelin(102)	Quinones	*Embelia ribes* Burm.f.	C4-2B	*In vitro*	Antiproliferation Induction of apoptosis	Induction of G1 cell cycle arrest; induction of apoptosis by triggering caspase 3 activation and PARP cleavage; inhibition of survivin expression by inhibiting AKT/NF-κB pathway.	Xu et al. (2017)
			DU145				
			PC3				
ESK246(103)	Glycosides	*Pittosporum venulosum*	LNCaP	*In vitro*	Antiproliferation	Inhibition of leucine uptake, leading to reduced mTORC1 signaling, cell cycle protein expression and cell proliferation.	Wang et al. (2014)
Ginsenoside Rg3(104)	Steroids	*Panax ginseng* C.A.Mey.	PC-3M	*In vitro*	Antiproliferation	Suppression of aquaporin 1 (AQP1) water channel protein expression by activating p38 MAPK.	Pan et al. (2012)
					Antimetastasis		
Guggulsterone(105)	Steroids	*Commiphora mukul* (Hook. ex Stocks) Engl.	DU145 LNCaP PC3	*In vitro*	Antiproliferation Induction of apoptosis	Increase of Bax expression, downregulation of Bcl-xl and Bcl-2 expression; increase of caspase-9 and caspase-8 cleavage; increase of ROI generation by activating JNK; selective inhibition of androgen receptor promoter activity in LNCaP cell.	Singh et al. (2005)
			PrEC				
Guttiferone F(106)	Prenylated benzophenones	*Allanblackia stuhlmannii* (Engl.) Engl.	LNCaP PC3	*In vitro*	Induction of apoptosis	Increase of sub-G1 fraction and DNA fragmentation; down-regulation of androgen receptor expression and phosphorylation of ERK1/2.	Li et al. (2015c)
				*In vivo*			
Indole-3-carbinol (I3C)(107)	Indoles	Distributed in various plants	LNCaP	*In vitro*	Antiproliferation	Induction of G1 cell-cycle arrest and downregulation of AR expression and inhibition of AR promoter activity.	Hsu et al. (2005)
Juglone(108)	Quinones	*Juglans mandshurica* Maxim.	LNCaP	*In vitro*	Antiproliferation Induction of apoptosis	Downregulation of AR expression; increase of caspase-3 and -9 activity.	Jiang et al. (2013)
Marchantin M(109)	Diphenyls	*Asterella angusta*	DU145 LNCaP PC3	*In vitro*	Induction of apoptosis	Inhibition of the 20S proteasome activity; induction of microtubule-associated protein-1 light chain-3 beta (LC3B) expression and conversion; induction of RNA-dependent protein kinase-like ER kinase activity; suppression of the PI3K/AKT/mammalian target of rapamycin axis through preventing the activation and expression of AKT.	Jiang et al. (2013)
Moniliformediquinone (110)	Phenanthradiquinones	*Dendrobium moniliforme* (L.) Sw.	DU145 PC3	*In vitro*	Antiproliferation Induction of apoptosis Inhibition of tumor growth	Induction of S cell cycle arrest; induction of DNA damage response associated with Chk1, Chk2, c-Jun and JNK activation; induction of caspase-2,-3,7,8 and -9 cleavage through mitochondrial membrane loss and cytochrome c release.	Hsu et al. (2014)
				*In vivo*			
Oleuropein (111)	Glycosides	*Olea europaea* L.	LNCaP DU145 BPH-1	*In vitro*	Anti-oxidation Cytotoxicity	Not investigated.	Acquaviva et al. (2012)
				*In vivo*	Inhibition of tumor cell growth and invasiveness		
Physapubescin B (112)	Steroids	*Physalis pubescens* L.	PC3	*In vitro In vivo*	Antiproliferation	Downregulation of Cdc25C protein expression; induction of G2/M cell cycle arrest; decrease of Cdc25C level and increase of levels of CyclinB1, P21 and p-Cdk1 (Tyr15).	Ding et al. (2015)
Pristimerin (113)	Esters	Distributed in various plants	PC3	*In vitro*	Induction of apoptosis	Inhibition of proteasomal chymotrypsin-like activity assay and polyubiquitinated protein accumulation；Interaction with the proteasomal β5 subunit in a conformation suitable for proteasome inhibition; increase of caspase-3 activation.	Yang et al. (2008)
			LNCaP				
			C4-2B				
Phenethyl-Isothiocyanat(PEITC) (114)	Isothiocyan-ates	Distributed in various plants	C4-2B	*In vitro*	Antiproliferation	Increase in the G2-M phase; downregulate AR expression through inhibition of the transcription factor Sp1 and p300/CBP-associated factor (PCAF); upregulation of miR-194 via directly targeting BMP1, and downregulation of BMP1 led to decrease expression of key oncogenic matrix metalloproteinase, MMP2 and MMP9.	Wang et al. (2006), Yin et al. (2009), Jiang et al. (2013), Zhang et al. (2016)
			DU145 LNCaP PC3	*In vivo*	Induction of apoptosis		
Putrescine-1,4-dicinnamide (115)	Phenylprop-anoids	Distributed in various plants	DU145	*In vitro*	Induction of apoptosis	Increase the caspase-3 activity; increase of ROS generation.	Russo et al. (2007)
Physalins A(116)	Secosteriods	*Physalisalkekengi* var. *franchetii*	C4-2B 22Rv1	*In vitro*	Antiproliferation Induction of apoptosis	Inhibition of JNK and ERK activation; downregulation of AR expression and PAS expression.	Han et al. (2011)
Physalins B (117)	Secosteriods	*Physalisalkekengi* var. *franchetii*	C4-2B 22Rv1	*In vitro*	Antiproliferation	Inhibition of JNK and ERK activation; downregulation of AR expression and PAS expression.	Han et al. (2011)
					Induction of apoptosis		
Physachenolide C (118)	Steroids	Disbributed in various plants	LNCaP PC3	*In vitro*	Cytotoxicity Inhibition of tumor cell growth	Not investigated.	Xu et al. (2015)
				*In vivo*			
Reevesioside A (119)	Glycosides	*Reevesia formosana*	DU145 PC3	*In vitro*	Antiproliferation Induction of apoptosis	Induction of G1 cell cycle arrest by the downregulation of several related cell cycle regulators, including cyclin D1, cyclin E and CDC25A; increase of association between RB and E2F1 and the subsequent suppression of E2F1 activity via decreasing RB phosphorylation.	Leu et al. (2014)
Scopoletin(120)	Coumarins	*Erycibe obtusifolia*	LNCaP	*In vitro*	Antiproliferation	Induction of G2/M cell cycle arrest by the downregulation of cyclin D1 expression.	Li et al. (2015a)
					Induction of apoptosis		
Sulforaphane(SNF) (121)	Sulfides	Disbributed in various plants	TRAMP C1	*In vitro*	Anti-oxidation	Regulation of Nrf2’s CpGs demethylation and reactivation.	Zhang et al. (2013)
				*In vivo*			
Thymoquinone (122)	Quinones	*Nigella sativa*	C2-2B	*In vitro*	Induction of apoptosis	Not investigated.	Trang et al. (1993)
			DU145				
			LNCaP PC3				
Wedelolactone(123)	Esters	*Wedelia sinensis*	DU145 PrEC PC3	*In vitro*	Induction of apoptosis	Increase of c-JNK and caspase-3 activity by downregulation of PKCε without AKT inhibition.	Koka et al. (2010), Sarveswaran et al. (2012)
			LNCaP				
Z-Ligustilide(124)	Esters	*Angelica sinensis* (Oliv.) Diels	TRAMP C1	*In vitro*	Cytotoxicity	Increase of Nrf2 expression via the Nrf2 promoter CpGs demethylation.	Su et al. (2013)
2,5-dimethylphenol(125)	Phenols	*Chlaenius cordicolli*	PC3	*In vitro*	Cytotoxicity	Induction of [Ca^2+^]i rise through PKC-regulated store-operated Ca^2+^ channels and PLC- dependent Ca^2+^ release from the endoplasmic reticulum.	Wang et al. (2016a)
3-oxo-tirucallic acid(126)	Lupanic acids	*Boswellia carteri* Birdw*.*	LNCaP PC3	*In vitro*	Induction of apoptosis Inhibition of tumor cell growth	Inhibition of Akt activity and Akt signaling pathways, including glycogen synthase kinase-3β and BAD phosphorylation, and nuclear accumulation of p65, androgen receptor, β-catenin, and c-Myc.	Estrada et al. (2010)
				*In vivo*			
3-α-acetoxy-tirucallic acid(127)	Lupanic acids	*Boswellia carteri* Birdw*.*	LNCaP PC3	*In vitro*	Induction of apoptosis Inhibition of tumor cell growth	Inhibition of Akt activity and Akt signaling pathways, including glycogen synthase kinase-3β and BAD phosphorylation, and nuclear accumulation of p65, androgen receptor, β-catenin, and c-Myc.	Estrada et al. (2010)
				*In vivo*			
3-β-acetoxy-tirucallic acid(128)	Lupanic acids	*Boswellia carteri* Birdw*.*	LNCaP PC3	*In vitro*	Induction of apoptosis Inhibition of tumor cell growth	Inhibition of Akt activity and Akt signaling pathways, including glycogen synthase kinase-3β and BAD phosphorylation, and nuclear accumulation of p65, androgen receptor, β-catenin, and c-Myc.	Estrada et al. (2010)
				*In vivo*			
6α-acetoxyanopterine(129)	Esters	*Anopterus macleayanus*	LNCaP	*In vitro*	Antiproliferation	Interaction with tubulin.	Levrier et al. (2016)
			PC3		Induction of apoptosis		
24-epibrassinolide(130)	Brassinoste-roids	Distributed in various plants	DU145 LNCaP	*In vitro*	Antiproliferation;	Induction of G1 cell cycle arrest accompanied by reductions in cyclin D1, CDK4/6 and pRb expression in LNCaP cells; induction of G2/M cell cycle arrest by reductions in cyclin A, cyclin expression in DU145 cells.	Steigerova et al. (2012)
				*In vivo*	Induction of apoptosis		
25-OH-PPD (131)	Saponins	*Panax ginseng* C.A.Mey.	LNCaP PC3	*In vitro*	Antiproliferation	Induction of G1 cell cycle arrest by downregulation of MDM2, E2F1, Bcl2, cdk2/4/6, and cyclin D1 expressions; increase of p21, p27, and Bax expressions; induction of PARP cleavage and caspases activation.	Wang et al. (2008a)
					Induction of apoptosis		
				*In vivo*	Inhibition of tumor growth		
28-homocastasterone(132)	Brassinosteroids	Distributed in various plants	DU145 LNCaP	*In vitro*	Antiproliferation;	Induction of G1 cell cycle arrest accompanied by reductions in cyclin D1, CDK4/6 and pRb expression in LNCaP cells; induction of G2/M cell cycle arrest via reductions in cyclin A expression.	Steigerova et al. (2012)
				*In vivo*	Induction of apoptosis		
2′- Hydroxycinnamaldehyde (133)	Aldehydes	Cinnamomum verum J.Presl	DU145; LNCaP	*In vitro*	Antiproliferation;Induction of apoptosis	Signal transducer and activator of transcription 3 inactivation and reactive oxygen species generation	Yoon et al. (2019)
				*In vivo*			
Methyllucidone (134)	cyclopentenedione	*Lindera erythrocarpa* Makino (Lauraceae)	DU145	*In vitro*	Inhibition of cell growth; Induction of apoptosis	Arrest of the cell cycle at G1 phase;regulation of the expression of the protein tyrosine phosphatase MEG2	Jin et al. (2018)
Ophiopogonin D＇(135)	triterpenoid saponins	Radix Ophiopogonis	PC3 ； DU145	*In vitro*	Induction of apoptosis	Via a RIPK1-related pathway	Lu et al. (2018)
				*In vivo*			
Corchorusoside C(136)	steroid	*Streptocaulon juventas* (Lour.) Merr. (Apocynaceae)	DU-145	*In vitro*	Induction of apoptosis	Inhibition of activity and protein expression of NF-κB (p50 and p65), IKK(α and β), and ICAM-1;decrease of protein expression of Bcl-2 and increase of expression of PARP-1;increase of caspases 3 and 7	Anaya-Eugenio et al. (2019a)
				*In vivo*			
Daucosterol (137)	steroid saponin	*Crateva adansonii* DC (Capparaceae)	LNCaP;DU145;PC3	*In vitro*	Antiproliferation;inhibition of cell growth;Induction of apoptosis	Downregulation of cell cycle proteins (cdk1, pcdk1, cyclin A and B) in DU145 and PC3 cells;downregulation of cdk2 in PC3 cells;downregulation of Akt, pAKT 18 and Bcl-2 proteins;up-regulation of Bax	Zingue et al. (2019)
Cyanidin 3-glucoside (138)	Glycosides	The dark purple glutinous rice (Oryza sativa L.) cultivar Luem Pua (LP)	PC3	*In vitro*	Inhibition of progressive cancer cell behaviors	Inhibit EMT through Smad signaling pathway(s) mediating Snail/E-cadherin expression	Jongsomchai et al. (2020)
Germacrone (139)	Ketones	Rhizoma Curcuma	PC-3;22RV1	*In vitro*	Antiproliferation;Induction of apoptosis	Inhibiting the Akt/mTOR signaling pathway	Yu et al. (2020)
Ineupatolide (140)	Esters	*Carpesium cernuum* L.	PC-3	*In vitro*	Antiproliferation	Promoting apoptosis and arresting the cell cycle in the G2and S phases;	Huang et al. (2021)
Jegosaponin A and B (141 and 142)	Saponins	*Styrax japonica* Siebold et al. Zuccarini.	PC-3	*In vitro*	Exhibiting cell membrane disruptive properties	Not investigated	Nishimura et al. (2021)
				*In vivo*			
Isolobetyol (143)	polyacetylene	*Platycodon grandiflorus* (Jacq.) A.DC.	PC-3	*In vitro*	Antiproliferation	Not investigated	Li, (2020)
Lobetyol (144)	Alkynes	*Platycodon grandiflorus* (Jacq.) A.DC.	PC-3	*In vitro*	Antiproliferation	Not investigated	Li, (2020)
Lobetyolin (145)	Alkynosides	*Platycodon grandiflorus* (Jacq.) A.DC.	PC-3	*In vitro*	Antiproliferation	Not investigated	Li, (2020)

## Extracts With Anti-Prostate Cancer Activity

Extracts consist of a group of bioactive natural compounds, which may exert and possess the advantages of synergistic effects against diseases. Recently, nutraceuticals have also received increasing attention as the agents or dietary supplements for cancer prevention and treatment, as well as some extracts derived from edible sources. Thus in this section we will respectively review those extracts and nutraceuticals that have the potential effects against prostate cancer either *in vitro* or in prostate cancer mice models. Chinese herbal compound preparations of more than one medicinal plants that have been reported to inhibit prostate cancer are also presented in this review.

### Herbal Extracts

Traditional and folk herbal medicines from medicinal plants offer great potential for the discovery of novel anti-prostate cancer drugs. The plant extracts listed in Table 8 are complex mixtures, which need further investigations to reveal their bioative constituents through bioguided isolation and to clarify the roles of these different compounds in agaisnt prostate cancer when used alone or in combination. Also, the synergistic effect of the individual active components of these extracts and molecular mechanisms involved need further elucidation in order to evaluate the potential of these compounds as antineoplastic agents.

**TABLE 8:** Extracts obtained from plants with anti-prostate cancer activities.

Extract	Botanical name	Medicinal part	Cell type	Observation	Activity	Mechanism of action	Refs
Ethanol extract	*Vitex negundo* L.	Seed	PC3	*In vitro*	Antiproliferation	Induction of cleavage in poly ADP ribose polymerase protein; upregulation of Bax and downregulation of Bcl-2; increase of caspase-3 and -9.	Zhou et al. (2009)
				*In vivo*			
Methanolic extract	*Aloe perryi* Baker	Fruit	HTB-81	*In vitro*	Induction of apoptosis	Not investigated.	Al-Oqail et al. (2016)
Ethanolic extract	*Annona muricata* L.	Leaf	PC3	*In vitro*	Antiproliferation	Not investigated.	Yang et al. (2015)
				*In vivo*	Inhibition of tumor growth		
Aqueous extract	*Camellia sinensis* (L.) Kuntze	Leaf	PC3	*In vitro*	Antiproliferation	Increase of Bax/Bcl-2 ratio and decrease of Ki67 protein expression; decrease of blood concentrations of tumor growth factors and tumor concentrations of VEGF and EGF expressions.	Wang et al. (2016b)
				*In vivo*	Induction of apoptosis Inhibition of tumor growth		
Methanolic extract	*Artocarpus altilis* (Parkinson) Fosberg	Leaf and stem	DU145 LNCap PC3	*In vitro*	Antiproliferation	Inhibition of STAT3 Ty705 phosphorylation and STAT3 activation.	Jeon et al. (2015a)
					Induction of apoptosis		
				*In vivo*	Inhibition of tumor growth		
Hexane extract	*Juglans regia* L.	Leaf	PC3	*In vitro*	Antiproliferation	Not investigated.	Li et al. (2015b)
					Induction of apoptosis		
Ammonia dichloromethane extract	*Berberis libanotica*	Root	DU145 PC3 22Rv1	*In vitro*	Antiproliferation	Induction of G0/G1 cell cycle arrest; eradication of self-renewal ability of highly resistant prostate cancer stem cells.	
					Inhibition of metastasis		
Supercritical extract	*Azadirachta indica* A.Juss*.*	Leaf	LNCaP PC3	*In vitro*	Antiproliferation	Inhibition of calreticulin, integrin b1, and focal adhesion kinase activation; increase of the AKR1C2 level.	Wu et al. (2014)
				*In vivo*	Induction of apoptosis Inhibition of tumor growth		
Acetone extract	*Chaenomeles japonica* (Thunb.) Lindl. ex Spach	Fruit	PNT1A PC3	*In vitro*	Induction of apoptosis	Increase of Bax/Bcl-2 ration.	Lewandowska et al. (2013)
Hexanic lipidosterolic extract	*Serenoa repens* (W.Bartram) Small	Whole plant	LNCaP PC3	*In vitro*	Induction of apoptosis	Increase of caspase 9 activation and poly (ADP-ribose) polymerase 1 cleavage, and mitochondrial PTP activation.	Baron et al. (2009)
Aqueous extract	*Taraxacum officinale* F.H.Wigg.	Root	C4-2B LNCaP	*In vitro*	Inhibition of metastasis	Decrease of phosphorylation levels of FAK and SRC, and activities of matrix metalloproteinases (MMP-2 and MMP-9).	Sigstedt et al. (2008)
Ethyl acetate extract	*Commiphora mukul* (Hook. ex Stocks) Engl.	Bark	LNCaP PrEC	*In vitro*	Induction of apoptosis	Decrease of Bax expression; suppression of JNK activation.	Xiao et al. (2011)
Aqueous extract	*Cistus creticus* L. ； *Cistus monspeliensis* L.	Whole plant	PZ-HPV-7 PNT1A	*In vitro*	Antiproliferation	Not investigated.	Vitali et al. (2011)
Acidified dimethyl sulfoxide extract	*Morus nigra* L.	Fruit	PC3 CRL1435	*In vitro*	Antiproliferation	Induction of G1 cell cycle arrest; decrease mitochondrial membrane potential.	Turan et al. (2017)
					Induction of apoptosis		
Dichloromethane extract	*Strobilanthes crispa* (L.) Blume	Leaf	DU145 PC3	*In vitro*	Induction of apoptosis	Increase of caspase 3 and/or 7 activity.	Visweswaran et al. (2010)
Punica granatum exeract	*Punica granatum* L.	Seed	LNCaP PC3	*In vitro*	Induction of apoptosis Inbition of metastasis	Upregulation of p21 and p27; increase of JNK phosphorylation; suppression of AKT/mTOR signaling; modulation of the IGF-IGFBP axis.	Deng et al. (2017)
Hydroalcoholic extract	*Justicia spicigera* Schltdl.	stems;	LNCaP	*In vitro*	Antiproliferation	cytostatic mechanism	Fernández-Pomares et al. (2017)
		leaves					
Ethyl acetate fraction	*Phoenix dactylifera* L. (Ajwa dates)	Fruit	PC3	*In vitro*	Induction of apoptosis	Arrest of the cell cycle in S phase	Mirza et al. (2018)
Ethyl acetate extract	*Kalanchoe flammea* Stapf (Crassulaceae)	Leaves	PC-3;	*In vitro*;	Induction of apoptosis	Phosphatidylserine translocation; overproduction of reactive oxygen species;release of Cytochrome C at mitochondrial level; activation of caspase-3 and -9;downregulation of apoptosis-related proteins Bcl-2, XIAP, and PKCε and DNA fragmentation and cell cycle arrest	Arias-González et al. (2018)
			LNCaP;				
			PrEC	*In vivo*			
Hydroalcoholic extracts	*Euphorbia szovitsii* Fisch. & C.A.Mey., U. dioica and *Medicago sativa* L.	aerial parts	PC-3, DU145 ； HDF	*In vitro*	Antiproliferation	Not investigated	Asadi-Samani et al. (2018)
Ethanol extract	*Moringa oleifera flower*	flower	PC-3	*In vitro*	Induction of Apoptosis	Downregulation of AKT Pathway	Ju et al. (2018)
methanolic extract	*Paederia foetida* L.	leaves	PC-3, DU-145,HaCaT	*In vitro*	Inhibition of cell growth, migration; induction of apoptosis	Modulating chromatin modification enzymes and altering pro-inflammatory cytokine gene expression	Pradhan et al. (2019)
Ethanolic extract	*Moringa peregrina* (Forssk.) Fiori	seed	PC-3	*In vitro*	Induction of apoptosis	Cell cycle arrest at sub-G0 phase and DNA fragmentation.	Abou-Hashem et al. (2019)
Ethanolic extract	*dandelion* (Taraxacum officinale) *root* and *lemongrass* (Cymbopogon citratus)	Root		*In vivo*	Induction of apoptosis	Not investigated	Nguyen et al. (2019)
Ethanolic extract	*Glycyrrhiza glabra* (Fabaceae family)	roots	PC-3	*In vitro*	Antiproliferation	Both apoptosis and autophagy mechanisms	Gioti et al. (2020)
Dimethyl sulfoxide extract	*Ganoderma lucidum*	whole plant	PC3	*In vitro*	Induction of apoptosis	Inhibition of Jak-1/STAT-3 activity	Wang et al. (2020)
Methanolic extract	*Moringa oleifera* Lam.	leaf	DU145;PC-3	*In vitro*	Antiproliferation; induction of apoptosis;G0/G1 cell cycle arrest	Downregulation of Notch signaling；downregulation of Hedgehog Signaling Pathway	(Khan et al., 2020a; Khan et al., 2020b)
Dichloromethane and methanol extract	*Cecropia pachystachya* Trécul	leaves	PC3	*In vitro*	Senescence induction	β-galactosidase overexpression	Rosa et al. (2020)
Methanolic extract	*Dracocephalum palmatum* Stephan	leaves	PC-3	*In vitro*	Induction of apoptosis	Via the caspase-8-mediated extrinsic pathway	Lee et al. (2020)
Extract of multi-solvent systems(Cyclohexane, Hexane, Diethyl Ether, Ethyl Acetate, Methanol, Water)	*Hippophae rhamnoides* L. and *Hippophae tibetana* Schltdl.	leaves	LNCaP;C4-2	*In vitro*	Antiproliferation	Downregulation of androgen responsive genes, PSA, ELL2, EAF2 and CALR	Masoodi et al. (2020)
Ethanolic extract	*Salvia miltiorrhiza* Bunge.	roots	DU-145	*In vitro*;	Antiproliferation;induction of apoptosis;	Increase of the expression of p53 and reducion of the expression of Bcl-2 proteins	Bae et al. (2020)
				*In vivo*			
Ethanolic extract	*Spirogyra neglecta* (Hassall) Kutzing	whole plant	PC3	*In vitro*	Antimetastasis activity	Inhibiting the Akt signaling pathway	Arjsri et al. (2021)
Methanolic extract	*Artemisia kruhsiana* subsp. alaskana (Rydb.) D.F.Murray & Elven	stems and leaves	PC-3	*In vitro*	Induction of autophagy;	Inhibitions of phosphor (p)-AKT, p-mTOR, Bcl-2, and Bax, activating beclin 1 and LC3 ratio in PC-3 cells	Lee et al. (2021)
Ethanolic extracts	*Treculia africana* Decne. (Moraceae) and *Entandrophragma angolense* Welw (Meliaceae)	whole plants	LNCaP, DU145 and PC3	*In vitro*	Antiproliferation;induction of apoptosis; anti-metastatic effects	Overexpression of caspase-3; low expression of Akt, pAkt and Bcl-2 proteins;a decrease of chemotaxis and cell migration	Zingue et al. (2021)
Ethanolic extracts	*Moringa oleifera* Lam.	Flower	PC-3	*In vitro*	Induction of apoptosis	Downregulation of AKT Pathway	Ju et al. (2018)

### Chinese Herbal Compound Preparations

There are four traditional Chinese medical formulations reported to display significant anti-prostate cancer properties, that is, Zyflamend, PC-SPES and LCS101, which are composed of different medicinal plants (Table 9; Bemis et al., 2005; Hsieh et al., 1997; Cohen et al., 2015). Especially, PC-SPES significantly inhibited prostate tumor growth in tumor-bearing mouse model, mainly through cell cycle arrest and apoptosis induction, which is already clinically utilized for the treatment of clincal patients with prostate cancer (Hsieh et al., 1997).

**TABLE 9:** Chinese herbal compound preparations obtained from plants with anti-prostate cancer activities.

Medical formulation	Composition	Cell type	Observation	Activity	Mechanism of action	Refs
Zyflamend	Rosemary; Ginger; Turmeric; Green tea; Holy basil; Hu zhang; Chinese goldthread;	LNCaP	*In vitro*	Antiproliferation	Inhibition of COX-1 and COX-2 enzyme activities; upregulation of p21 expression; downregulation of AR expression; induction of phosphorylation of Stat3 and PKCα/β.	Bemis et al. (2005)
	Oregano; Barberry;					
	*Scutellaria baicalensis.*			Induction of apoptosis		
PC-SPES	*Ganoderma lucidium;*	DU145	*In vitro*	Induction of apoptosis	Induction of G0/G1 and G2/M cell cycle arrest; upregulation of p21waf1 expression and downregulation of Bcl-2 expression.	Hsieh et al. (1997)
	*Dendranthema morifolium; Isatis indigotica; Glycyrrhiza glabra; Rabdosia rubescens; Panax pseudoginseng; Serenoa repens; Scutellaria baicalensis;.*	LNCaP	*In vivo*	Inhibition of tumor growth		
		PC3				
LCS101	*Atractylodes macrocephala; Astragalus membranaceus; Glehnia littoralis; Citrus reticulate; Lycium chinense; Ligustrum lucidum; Oldenlandia diffusa; Milletia reticulata; Ophiopogon japonicus; Paeonia obovata; Paeonia lactiflora; Prunella vulgaris; Poriae cocos; Scutellaria barbata.*	DU145	*In vitro*	Induction of apoptosis	Not investigated	Cohen et al. (2015)
		PC3				

### Nutraceuticals and Extracts Derived From Edible Sources

Nowadays, dietary factors play an increasingly important role in the chemopreventive and/or therapeutic management of cancer (Table 10). The study of dietary agents (nutraceuticals or extracts derived from edible sources) in prostate cancer prevention is an important area of research since about 43–80% patients with prostate cancer are on alternative therapy based on dietary modification (Lippert et al., 1999; Nam et al., 1999). There are strong evidences that nutraceuticals and extracts derived from edible spices, vegetables or fruits such as vitamin D, pomegranate and tea polyphenols have demonstrated significant anti-prostate cancer activity when tested either *in vitro* and/or *in vivo* (Kasimsetty et al., 2009; Gregory et al., 2010; Koyama et al., 2010; Mordanmccombs et al., 2010; Hsu et al., 2011; Xiao et al., 2011; Turan et al., 2017). Especially, dietary phytochemicals that can selectively interfere cellular pathways involved in prostate cancer cells have attracted research interest of scientists in prostate cancer therapies in recent years.

**TABLE 10:** Nutraceuticals and extracts obtained from plants with anti-prostate cancer activities.

Dietary agents	Cell type	Observation	Activity	Mechanism of action	Refs
Epigallocatechin-3-Gallate (Green Tea)	Du145	*In vivo*	Antiproliferation	Downregulation of ID2; increase of Bax/Bcl-2; inducing cell death via an ID2-related mechanism; Antiproliferation by increasing the activity of ERK 1/2 through a MEK-independent and PI3K-dependent mechanism.	Xiao et al. (2011), Turan et al. (2017)
	LNCaP	*In vitro*	Induction of apoptosis		
Grape skin	C4-2B LNCaP ARCaP-E	*In vitro*	Induction of apoptosis Inhibition of metastasis	Decrease of nail and pSTAT3 expression; inhibition of Snail-mediated CatL activity.	Burton et al. (2015)
		*In vivo*			
Modified Citrus Pectin	LNCaP PC3	*In vivo*	Induction of apoptosis	Cell growth inhibition and apoptosis induction via inhibiting MAPK/ERK signaling pathway and activating caspase 3.	Azémar et al. (2007), Yan and Katz (2010)
		*In vitro*			
ProstaCaid	PC3	*In vitro*	Antiproliferation	Downregulation of expression CCND1, CDK4, E2F1and MAPK6; upregulation of CDKN1A expression; downregulation of CAV1, IGF2, NR2F1, and PLAU genes expression; suppression of the urokinase plasminogen activator (uPA) secretion.	Jiang et al. (2011)
			Inhibition of and migration invasion		
Pomegranate	C4-2B	*In vitro*	Antiproliferation	Inhibition of enzyme (cytochrome P450) expression and activity; inhibition of mTOR phosphorylation at Ser2448 and Ser2481 and IGF1 expression.	Malik et al. (2005), Hong et al. (2008), Kasimsetty et al., (2009), Koyama et al. (2010)
	DU145				
	LNCaP				
	LNCaP-AR	In vivo	Induction of apoptosis Inhibition of metastasis		
	PC3				
	22Rv1				
Selenium	C4-2B LNCaP PC3	*In vitro*	Antiproliferation	Increase of p53 expression; apoptosis induction by superoxide generation through the mitochondrial-dependent pathway	Pinto et al. (2007), Xiang et al. (2009), Sarveswaran et al. (2010)
		*In vivo*	Induction of apoptosis		
Soy	LNCaP	*In vitro*	Antiproliferation	Decreased of COX-2 RNA and protein expression; inhibition of the synthesis of prostaglandins; downregulation growth factors involved in angiogenesis (EGF and IGF-1) and the IL-8 gene; inhibition of ERK-1 and ERK-2 expression.	Wang et al. (2004), Swami et al. (2009), Rabiau et al. (2010)
	PC3	*In vivo*	Inhibition of tumor growth		
Vitamin D	DU145 LNCaP PC3	*In vitro*	Antiproliferation	Increase of E-cadherin expression; decrease of urokinase plasminogen activator receptor levels.	Gregory et al. (2010), Mordanmccombs et al. (2010), Hsu et al. (2011)
		*In vivo*	Inhibition of migration		
Vitamin K2	VCaP	*in vitro*	Antiproliferation;Induction of apoptosis	Downregulation of the expression of androgen receptor, BiP, survivin, while activating caspase-3 and -7, PARP-1 cleavage, p21 and DNA damage response marker, phospho-H2AX	Dasari et al. (2018)
Algerian propolis	LNCaP	*in vitro*	Induction of apoptosis	Blocking the cell cycle at G0/G1 phase.	Zabaiou et al. (2019)

## Conclusion and Perspectives

Prostate cancer is the second most frequently diagnosed tumor and the fifth leading cause of cancer-related deaths in men in the worldwide (McEleny et al., 2002). And the mortality of prostate cancer mainly occurs as a result of the castrate resistant ones. Up to date, different kinds of drugs have been employed to improve the treatment condition, mainly including LHRH antagonists, antiandrogen (androgen receptor antagonists and androgen synthesis inhibitors), tyrosine kinase inhibitors, angiogenesis inhibitors, endothelin antagonists, matrix metalloproteinase inhibitors, antioxidants, and cell cycle inhibitors. However, as mentioned above, there is no effective therapy for CRPC at present, except for docetaxel, which is the only chemotherapeutic agent that has been proven to prolong the overall survival in CRPC patient population though with many adverse effects reported (Eyben et al., 2015). Hence, it is urgent for us to explore an effective treatment for prostate cancer, especially for CRPC. In recent years, many natural products and extracts have been scientifically investigated *in vitro* and/or *in vivo* and proved as potential anti-prostate cancer agents, which are currently scattered across various publications. So a systematic summary and knowledge of future prospects are necessary to facilitate further chemical and pharmacological studies for anti-prostate cancer agents.

In our review, we provided a comprehensive overview of the molecular basis of the incidence and development of prostate cancer, especially for castration-resistant prostate cancer (CRPC), which mainly including canonical AR signaling (AR amplification, over-expression, mutation, and unconventional activation), and non-nuclear AR signaling (PI3K/AKT, Src, MAPKs, JAK-STAT3, and Ca^2+^ signaling pathways). So most components involved in above-mentioned pathways represent potential targets for screening natural compounds and/or extracts with anti-prostate cancer activity. And natural compounds or extracts that could function as modulators of canonical AR or non-nuclear AR signalling pathways thus can be regarded as promising candidates for anti-prostate interventions.

So far, a great amount of natural products isolated from diverse sources have been found to significantly inhibit prostate cancer cell proliferation/tumor growth or affected cellular signaling pathways in prostate cancer. As shown in our paper, the majority of natural compounds with direct relevance to prostate cancer are primarily derived from plants, with comparatively few molecules from marine and microbial sources. For these reported bioactive constituents, there is still plenty of room for improvement regarding the studies focused on efficacy enhancement and side effects amelioration by semi-synthetic modifications based on quantitative structural activity relationship elucidation. Since marine and microbial organisms represent important sources for single molecules exploit, more available and improved approaches should be included in finding novel natural products with significant anti-prostate cancer activity from these resource. Especially, engineering bacteria or fungus with novel gene clusters, currently used mainly for the identification of antibiotics or anti-tumor drugs, would be another promising approach for discovering natural compounds with anti-prostate activity. Extracts are another applicable option for anti-prostate purposes, in which case the chemical profile should be further elucidated, possibly affording a pure bioactive compound with precise mechanism of action. Also clinically used Chinese herb preparations should be profiled using techniques such as HPLC–MS to standardize the complex system to make it more controllable, stable, and reproducible in prostate cancer treatment. Furthermore, drug combination of these reported natural compounds with conventional chemotherapeutic agents may also be a promising way in finding solution for prostate cancer treatment. Finally, safty large-scale studies are needed to evaluate promising compounds or extracts and determing non-toxic doses for treating prostate cancer in mammals.

In conclusion, tackling prostate cancer (especially CRPC) is a much needed task that requires not only the great progress in understanding the genetic basis of prostate cancer, but also the significant technological improvements in tracking of bioactive natural compounds and structural characterization, which will facilitate the identification of novel natural compounds with significant anti-prostate cancer properties for drug development and therefore can be translated into significant health benefits for humans.
